# Targeting conserved interaction surfaces of alphavirus nsP4 supports the rational design of live-attenuated vaccine candidates

**DOI:** 10.1128/mbio.01638-26

**Published:** 2026-09-10

**Authors:** Xiaoyao Yang, Evelin Väljaots, Kaiyu Li, Krystyna Naumenko, Sandra Koit, Yaw Bia Tan, Xiang Liu, Victor Rodrigues de Melo Costa, Suresh Mahalingam, Dahai Luo, Andres Merits, Jian Yang

**Affiliations:** 1 Institute of Basic Medicine, North Sichuan Medical College 74655, Nanchong, People’s Republic of China; 2 Institute of Bioengineering, University of Tartu 37546, Tartu, Estonia; 3 Lee Kong Chian School of Medicine, Nanyang Technological University 54761, Singapore, Singapore; 4 School of Pharmacy and Medical Sciences, Griffith University, Gold Coast, Australia; 5 Institute for Biomedicine and Glycomics, Griffith University, Gold Coast, Australia; Duke University School of Medicine, Durham, North Carolina, USA

**Keywords:** alphavirus, RNA virus, viral replication, vaccines, RNA replication, RNA polymerases, virulence

## Abstract

Alphaviruses are emerging arboviruses that pose a growing threat to global public health; however, effective interventions remain limited. Recent structural studies of the chikungunya virus (CHIKV) replicase core revealed that the viral RNA-dependent RNA polymerase nsP4 interacts with both nsP1 and nsP2 and is predicted to engage viral RNA. The conservation and multifunctionality of nsP4 make it a challenging but promising target for rational attenuation. Here, we evaluated 58 structure-guided mutations in nsP4 predicted to disrupt interactions with nsP1, nsP2, or viral RNA using a *trans*-replicase system. Sixteen selected mutations were further analyzed in a CHIKV-o’nyong-nyong virus chimera and Semliki Forest virus (SFV). Most mutants were viable but showed varying degrees of attenuation in mammalian and mosquito cells. Viruses carrying mutations in predicted RNA-interaction sites frequently acquired pseudoreversions and/or second-site compensatory changes, whereas those harboring mutations affecting nsP1 or nsP2 interfaces were more genetically stable. Three substitutions representing defects in nsP1 interaction (F164A), predicted RNA interaction (K174S), and nsP2 interaction (D393A) were further characterized in authentic CHIKV and displayed attenuation consistent with that observed in SFV. *In vivo*, all three variants showed reduced pathogenicity in the SFV mouse model, with F164A being avirulent, immunogenic, and conferring complete protection against lethal challenge. In a CHIKV mouse model, these mutations attenuated disease and reduced serum virus titers and inflammatory cell infiltration. Together, these findings provide a molecular framework for the rational design of live-attenuated alphavirus vaccine candidates and support broader evaluation of nsP4-based attenuation.

**IMPORTANCE:**

Alphaviruses are an increasing global health concern, with hundreds of thousands of chikungunya cases reported worldwide in 2025; however, vaccine options remain limited, and no specific antiviral treatment is available. Recent structural studies have revealed conserved functional interfaces in the viral RNA polymerase nsP4, but their value for attenuation had not been systematically explored. Here, using structure-guided mutagenesis, validation in multiple viral systems, and *in vivo* analysis, we show that selected nsP4 mutations can strongly attenuate replication and pathogenicity while preserving immunogenicity. These findings identify conserved nsP4 interfaces as a shared vulnerability of alphaviruses and provide a molecular framework for the rational design of broadly applicable live-attenuated vaccines while also highlighting nsP4 as a functionally constrained target relevant to antiviral development.

## INTRODUCTION

Alphaviruses (family *Togaviridae*) are emerging, enveloped viruses with a positive-sense RNA genome (1). Most alphaviruses are mosquito-borne and capable of infecting a wide range of vertebrate hosts, including humans, horses, birds, and rodents (2). Based on genetic relatedness and clinical manifestations, alphaviruses are broadly classified into arthritogenic and encephalitic viruses. The arthritogenic group includes chikungunya virus (CHIKV), o’nyong-nyong virus (ONNV), Ross River virus (RRV), and Mayaro virus (MAYV), whereas Venezuelan, Western, and Eastern equine encephalitis viruses (VEEV, WEEV, and EEEV) represent the encephalitic group (3, 4). Driven by climate change, globalization, and the loss of wildlife habitats, alphavirus infections pose an increasing threat to public health and economic stability (5–9). In 2025, more than 500,000 confirmed and suspected CHIKV cases—including 186 fatalities—were reported worldwide, with nearly 30,000 cases in Guangdong and Guangxi Provinces (10), marking the largest recorded outbreak in China (11, 12).

Alphaviruses have evolved sophisticated mechanisms that enable them to cycle efficiently between human and mosquito hosts, complicating efforts to control transmission (13, 14). Over several decades, diverse vaccine platforms have been explored for CHIKV and related alphaviruses (15, 16). In 2023, the U.S. Food and Drug Administration (FDA) approved IXCHIQ, a live-attenuated CHIKV vaccine (17, 18), which was, however, later withdrawn from use following safety-related concerns (19). In February 2025, the virus-like particle (VLP)-based vaccine VIMKUNYA received FDA approval, representing a major milestone in CHIKV prophylaxis (20, 21). Despite these advances, no licensed vaccines exist for other alphaviruses, and there are currently no approved antiviral therapies for the treatment of alphavirus infections.

A major obstacle to developing vaccines and antivirals has been limited investment in fundamental research, the complexity of virus–host interactions, and the continual adaptive evolution of alphaviruses. The alphavirus genome is approximately 11.5 kb in length and contains two open reading frames (ORFs) that encode polyprotein precursors. The 5′ ORF is translated directly from the genomic RNA, whereas the 3′ ORF, which encodes the structural proteins, is translated from a subgenomic (SG) mRNA produced during infection (22). Translation of ORF1 yields the nonstructural (ns) polyproteins P123 and P1234, which are subsequently processed into four mature ns proteins (nsP1–nsP4), the subunits of the viral replicase complex (RC) (23). Alphaviral RNA replication is tightly regulated and dependent on the stepwise processing of P1234 by the protease activity of nsP2 (24–26), as well as on coordinated interactions among ns polyproteins, processing intermediates, mature nsPs, the genomic RNA, and host cell factors and structures (27–29). In infected cells, alphaviruses remodel the plasma membrane to generate invaginated membrane structures known as spherules, which serve as dedicated sites of viral RNA synthesis (30–34). The RC localizes at the neck of the spherules and consists of the membrane-embedded replicase core and associated cytosolic ring-like structure (35, 36). Recent studies have resolved the structure of the CHIKV replicase core, revealing a membrane-anchored dodecameric ring formed by nsP1, the viral RNA-capping enzyme. A single nsP4 molecule—the RNA-dependent RNA polymerase (RdRp) (37)—is positioned at the center of the ring, contacting multiple nsP1 subunits at the interior ring. The nsP2, which possesses protease, NTPase, RNA helicase, and RNA triphosphatase activities (38), associates with the cytosolic side of the replicase core complex and interacts with nsP4 to support replicase function (35).

nsP4 is the most conserved ns protein among alphaviruses and, in addition to its RdRp activity, also exhibits terminal adenylyltransferase activity essential for the synthesis of the poly(A) tails of genomic and SG RNAs (37–40). Its central role in viral RNA replication makes nsP4 an attractive target for antiviral intervention, and its manipulation holds promise for generating mutant viruses useful for biotechnology applications or as attenuated vaccine candidates (41–45). However, rational development of such tools has been limited by the sparse structural and functional information available for nsP4. Recombinant nsP4 has historically been difficult to study due to its extremely low solubility, propensity for aggregation, dynamic flexibility, and limited enzymatic activity, all of which have hindered detailed biochemical and structural analyses. Only in 2022 was the first three-dimensional structure of the nsP4 RdRp domain determined, revealing a canonical right-handed “fingers-palm-thumb” architecture (46). In contrast to nsP4 incorporated into the replicase core, isolated nsP4 contains several disordered regions. Most notably, the ~100-amino-acid N-terminal region—unique to alphavirus nsP4 proteins—is largely unfolded. Subsequent studies indicate that this region plays a critical role in replicase core assembly by forming stabilizing interactions between the nsP1 ring and the RdRp domain of nsP4 (35).

Here, we leveraged recent structural information on nsP4 and the alphavirus replicase core to perform structure-guided mutagenesis of critical regions of nsP4—specifically, sites implicated in interactions with nsP1 and nsP2, as well as residues lining the putative RNA-interacting channel. The impact of these mutations on RNA replication was assessed using *in vitro trans*-replication assays, which identified residues essential for replicase activity as well as positions where mutations reduced—but did not abolish—RNA synthesis. Corresponding mutations were introduced into the genomes of chimeric CHIKV constructs harboring nsP4 from ONNV and into Semliki Forest virus (SFV), a well-established model alphavirus. Analyses of infectivity and replication kinetics across multiple host cell types demonstrated broadly similar mutation-associated phenotypes among the different viruses, ranging from lethality to mild attenuation; in several cases, viral fitness was further modulated by pseudoreversions or second-site compensatory mutations. Finally, three viruses bearing selected nsP4 mutations were evaluated in murine models of SFV and CHIKV, revealing attenuation of *in vivo* pathogenicity. Given the conserved functional consequences of these mutations in the systems analyzed and their attenuation in animal models, targeting nsP4 represents a promising strategy for developing live-attenuated alphavirus vaccine candidates, although broader validation will be required.

## RESULTS

### Mutations in conserved nsP4 residues involved in interactions with nsP1 or nsP2 have distinct effects on viral RNA synthesis

**Fig 1:**
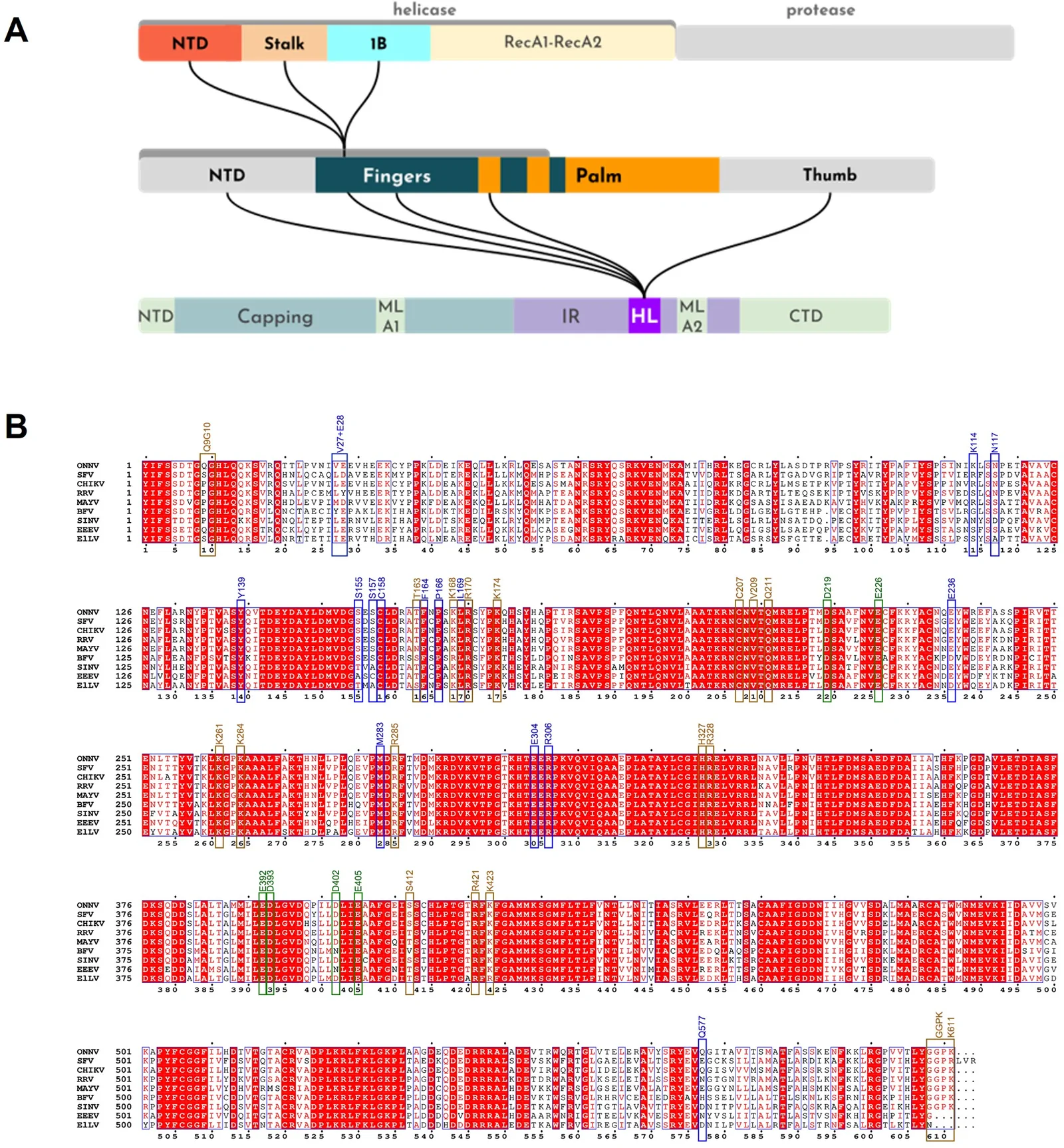
Conserved nsP4 residues involved in interactions with nsP1, nsP2, and RNA across alphaviruses. (**A**) Interaction map of nsP4 residues based on the resolved structure of the alphavirus replicase core. (**B**) Sequence alignment of nsP4 regions from representative alphaviruses, including members of the Semliki Forest antigenic complex—ONNV, SFV, CHIKV, RRV, and MAYV—as well as Barmah Forest virus (BFV), Sindbis virus (SINV), EEEV, and the insect-specific Eilat virus (EILV). White letters on a red background indicate fully conserved residues, red letters on a white background indicate similar residues, and black letters indicate non-conserved residues. nsP1-interacting residues analyzed in this study are marked and boxed in blue, nsP2-interacting residues are in green, and putative RNA-interacting residues are in brown. The alignment was generated using ESPript 3.

**Fig 2:**
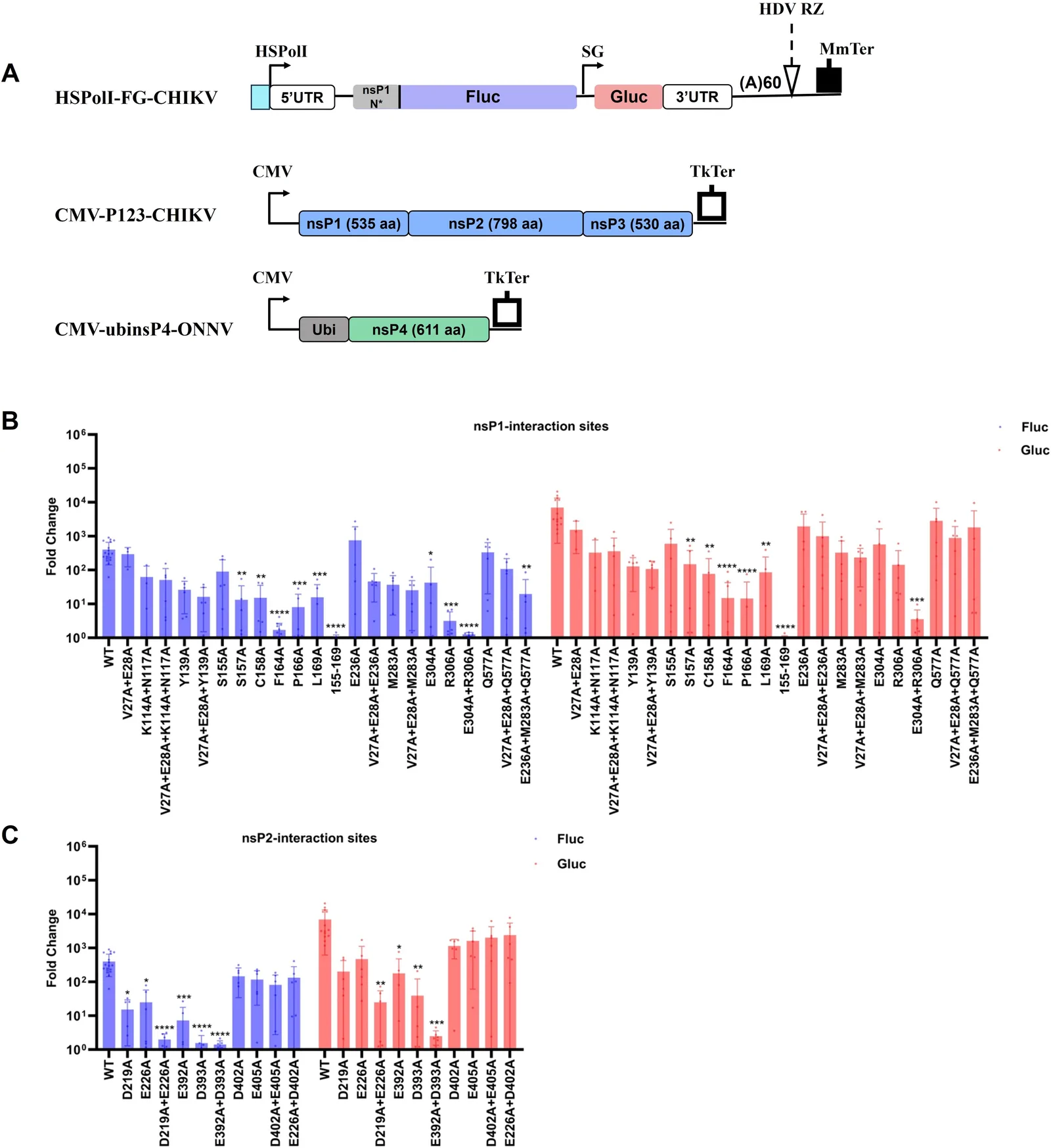
Mutations affecting conserved interactions within the replicase core attenuate alphavirus replicase activity. (**A**) Schematic representation of the template RNA (top) and replicase protein expression constructs. HSPolI, truncated human RNA polymerase I promoter (positions −211 to −1); 5′ UTR, full-length 5′ UTR of CHIKV; nsP1 N*, region encoding the N-terminal 77 amino acid residues of nsP1; SG, subgenomic promoter spanning position −79 to the end of the intergenic region relative to the nsP4 termination codon; 3′ UTR, truncated CHIKV 3′ UTR containing the terminal 110 nucleotides; HDV RZ, antisense-strand ribozyme of hepatitis delta virus; MmTer, mouse RNA polymerase I terminator; CMV, human cytomegalovirus immediate-early promoter; TkTer, herpes simplex virus thymidine kinase gene polyadenylation region; and Ubi, sequence encoding human ubiquitin. Vector backbones are not shown, and drawings are not to scale. (**B**) Functional impact of mutations in nsP1-interacting residues. (**C**) Functional impact of mutations in nsP2-interacting residues. (**B and C**) U2OS cells were cotransfected with HSPolI-FG-CHIKV encoding the mini-genome, CMV-P123-CHIKV, and CMV-ubinsP4-ONNV (wt) or the indicated mutant variants. In control cells, the plasmid expressing active nsP4 was replaced with the polymerase-inactive CMV-ubinsP4^GAA^ construct. Cells were lysed 18 hpi. Data represent luciferase activities (Fluc; replication, shown in blue, and Gluc; transcription, shown in pink) normalized to activities measured in CMV-ubinsP4^GAA^-transfected control cells. Individual data points and mean values ± standard deviation (SD) from at least three independent experiments are shown. Statistical analysis was performed using the Kruskal-Wallis test. *, *P* < 0.05; **, *P* < 0.01; ***, *P* < 0.001; ****, *P* < 0.0001.

To identify conserved nsP4 residues that contribute to interactions with nsP1 or nsP2, we utilized the high-resolution structure of the alphavirus replicase core composed of CHIKV nsP1, CHIKV nsP2, and ONNV nsP4 (35). Guided by this structure, we designed 22 mutations hypothesized to disrupt nsP1–nsP4 interactions and 10 mutations predicted to affect nsP2–nsP4 contacts (Fig. 1A; Table S1). All mutation sites mapped to regions highly conserved among alphaviruses (Fig. 1B). Corresponding single- and multiple-amino-acid substitutions were introduced into the nsP4 module of a two-component *trans*-replication system comprising CHIKV P123 and ONNV nsP4, a chimeric combination matching the replicase core for which the three-dimensional structure has been solved (35). In the presence of a CHIKV-derived mini-genome containing *cis*-acting elements required for interaction with the replicase complex and encoding firefly luciferase (Fluc) and *Gaussia* luciferase (Gluc) reporters (Fig. 2A), this system exhibits robust replicase activity and is well suited for the functional analysis of mutations (47). Replicase activities of the mutant complexes were quantified in human U2OS cells and compared to those of the wild-type (wt) complex. In this assay, enhancement of the Fluc signal (expressed as fold increase over the inactive nsP4^GAA^ control) reflects the synthesis of full-length “genomic” RNA, whereas enhancement of the Gluc signal reflects SG RNA production.

The mutations targeting nsP1–nsP4 interaction sites revealed both critical residues for this interaction and complex interplay among different contact points. Four single substitutions—S155A, E236A, E304A, and Q577A—as well as two double substitutions, V27A + E28A and K114A + N117A, had only minor effects on replication or transcription activities of the chimeric *trans*-replicase in human cells. Similar results were obtained when V27A + E28A was combined with either K114A + N117A or Q577A. However, when V27A + E28A was combined with E236A, a marked decrease in replicase activity was observed. These findings indicate that although these residues individually have limited impact on nsP4-dependent replicase function, functional impairment becomes evident when certain combinations of substitutions are introduced (Fig. 2B). In contrast, eight single mutations—Y139A, S157A, C158A, F164A, P166A, L169A, M283A, and R306A—prominently reduced replication and/or transcription. The most pronounced and highly significant defects were observed for mutants F164A and R306A, identifying F164 and R306 as key determinants of nsP4-dependent replicase function at the nsP1–nsP4 interface. (Fig. 2B). Combining F164A with S155A, S157A, C158A, P166A, and L169A completely abolished replicase activity, indicating additive or cumulative functional effects among these residues. Notably, addition of E304A to R306A further enhanced the loss of activity, suggesting a cooperative role for these charged residues. Similar synergistic effects were observed when V27A + E28A was combined with Y139A, and when E236A and Q577A were combined with M283A (Fig. 2B).

Mutations introduced into conserved residues at the nsP2–nsP4 interface also exhibited diverse functional effects. The D402A, E405A, and E226A each caused a relatively modest reduction of replicase activities. Corresponding combinations of these mutations also produced only minor effects (Fig. 2C). In contrast, the mutations at D219, E392, and D393 caused significant and pronounced reductions in replication and transcription activities. The combined E392A + D393A mutation resulted in a strong decrease in both replication and transcription, indicating a critical role for these adjacent charged residues. A similar but less pronounced effect was observed for the D219A + E226A combination. Collectively, these findings indicate that, like nsP1–nsP4 contacts, the nsP2-nsP4 interface function likely depends on cooperative contributions from multiple conserved residues within the interface.

### Mutations in nsP4 residues predicted to interact with viral RNA reduce *trans*-replicase activity

**Fig 3:**
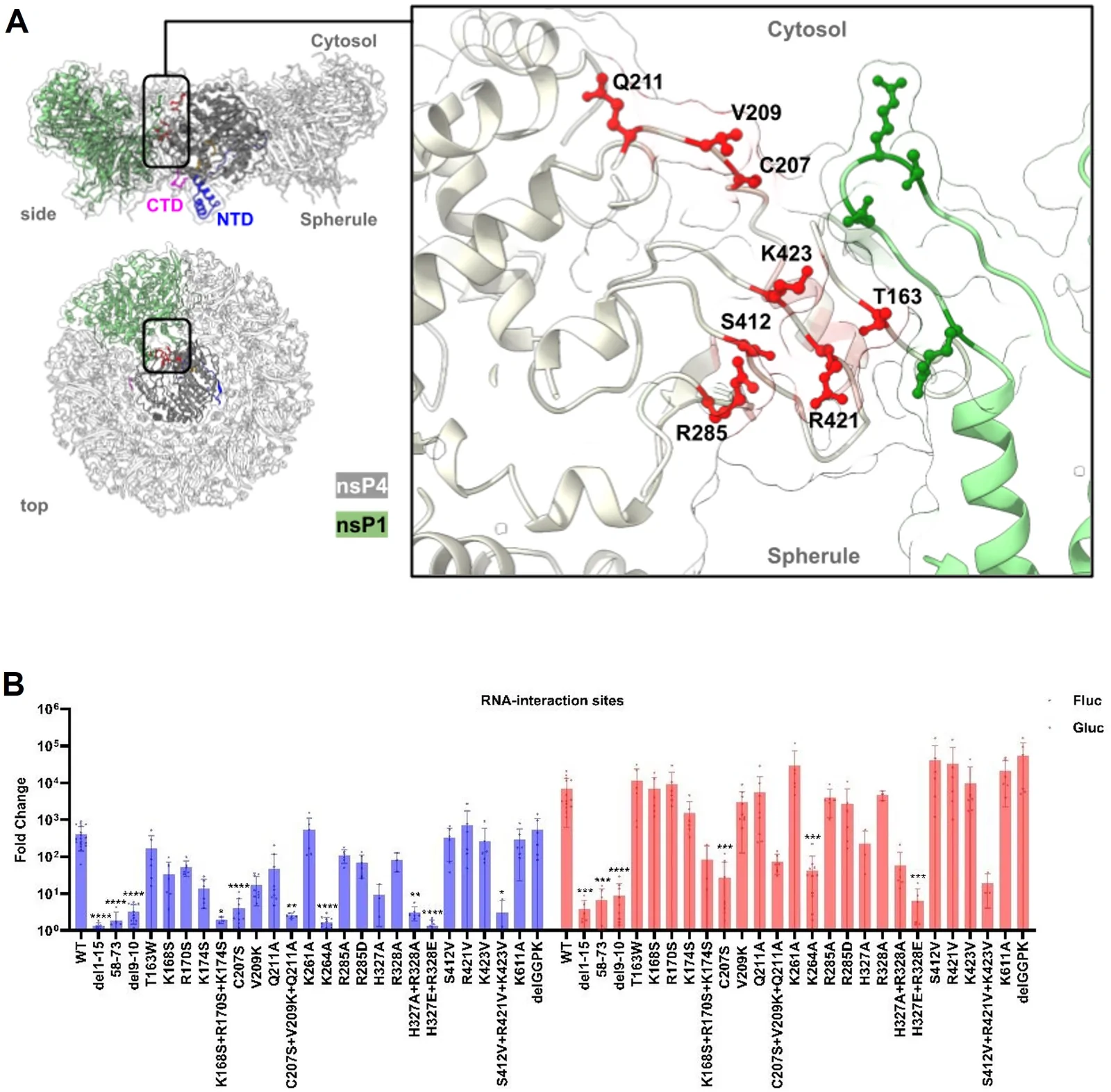
Mutations affecting putative nsP4-RNA interactions attenuate alphavirus replicase activity. (**A**) The putative RNA pore enabling the transport of viral RNA between the spherule interior and cytosolic space. Left panel: partial overview of RC core with nsP4 (gray) docked within the nsP1 dodecameric ring (chains A and B in green; chains C–L in white), shown in side and top views. Other nsP4 regions included in mutational analysis are shown in blue (NTD) and magenta (CTD). Right panel (zoomed): RNA-interacting residues of nsP4 within the pore analyzed by mutagenesis are highlighted in red. nsP1 chain A (inner-ring HL region) is shown in green, with residues 365, 369, 371, and 372 highlighted. (**B**) Functional impact of mutations in putative RNA-interacting residues. U2OS cells were cotransfected with HSPolI-FG-CHIKV encoding the template RNA, CMV-P123-CHIKV, and CMV-ubinsP4-ONNV (wt) or the indicated mutant variants. Experiments were performed, and data were collected as described for Fig. 2B and C. Individual data points and mean values ± SD from at least three independent experiments are shown. Statistical analysis was performed using the Kruskal-Wallis test. *, *P* < 0.05; **, *P* < 0.01; ***, *P* < 0.001; ****, *P* < 0.0001.

The mode of interaction between the alphavirus replicase core and viral RNA has not yet been fully experimentally resolved. To address this, available structural data (48) were combined with computational docking to predict nsP4 regions likely to participate in RNA binding. This analysis suggested that residues R285, S412, R421, and K423 line a putative RNA channel, whereas residues T163, C207, V209, and Q211 contribute to a guiding interface, that is, structural regions of nsP4 that may initially contact and “steer” a viral RNA (Fig. 3A). The RNA entry site itself is predicted to be shaped by positively charged residues, including K168, R170, and K174 (Fig. 3A). In addition, two pairs of closely positioned positively charged residues (K261/K264 and H327/R328), as well as C terminal K611, were expected to interact with RNA. Finally, the N-terminal region of nsP4 was predicted to contribute to RNA binding, potentially playing a role in priming RNA synthesis. In total, 26 mutations targeting these positions were introduced into the *trans*-replicase system (Fig. 1B; Table S2).

Deletion of the N-terminal 15 residues of nsP4 almost completely abolished CHIKV *trans*-replicase activity. A prominent reduction was also observed upon deletion of only two residues (Q9 and G10), as well as upon the replacement of putative RNA-interacting residues spanning positions 58–73 with valines (Fig. 3B). These findings support an essential role of the N-terminal region in viral RNA replication. In contrast, alanine substitution of the terminal residue K611, as well as deletion of the four C-terminal tail residues, had no significant effect, indicating that this region may be dispensable for RNA replicase activities (Fig. 3B).

A prominent reduction was detected in the mutant harboring the triple substitution S412V + R421V + K423V; notably, none of these three substitutions had a significant negative effect when introduced individually. In contrast, single substitutions R285A or R285D markedly reduced replication activity, highlighting the functional importance of residues within the putative RNA pore. Extending this analysis to the predicted guiding interface revealed a comparable pattern: except for T163W, all substitutions had a clear negative impact. Individually, C207S, V209K, and Q211A reduced replication activity, and their combination almost completely abolished it. Mutations in residues predicted to form the RNA entry site showed similar trends. Each of the single substitutions K168S, R170S, and K174S impaired replication activity, and combining all three resulted in a cumulative loss of function (Fig. 3B). Among mutations affecting two adjacent pairs of positively charged residues, K261A had no detectable effect, whereas K264A nearly eliminated replicase activity. Similar effects were observed for the second pair of charged residues: H327A caused a strong reduction, whereas R328A showed a more modest effect, and the double substitution produced a more severe phenotype. The importance of positive charge at this position was further supported by the complete loss of activity in the variant in which both residues were replaced with negatively charged glutamates (Fig. 3B).

### nsP4 mutations affecting *trans*-replicase activity also influence infectivity and growth of the CHIKV-ONNV chimeric virus

Although *trans*-replication systems are highly sensitive and informative, they remain model systems, and findings derived from them require validation in the context of viral infection. To accomplish this, we generated an infectious cDNA (icDNA) of a chimeric CHIKV genome in which the nsP4 region was replaced with that of ONNV (CHIKV^ONNVnsP4^), thereby matching the replicase composition used in the chimeric *trans*-replicase assays. Based on our previous observations that mutations with minimal effects in the *trans*-replicase system typically show no detectable impact on virus infection and that mutations causing complete or near-complete loss of *trans*-replicase activity are lethal in the viral context, we narrowed the panel of 58 tested mutations to those that produced measurable—but not complete—loss of replicase activity. Corresponding mutations were introduced into the CHIKV^ONNVnsP4^ genome, yielding a panel of mutant viruses for further analysis. This panel included four mutants with defects in nsP1 interaction, three mutants with impaired nsP2 interaction, and nine mutants affecting putative RNA-interaction sites (Table S3).

**Fig 4:**
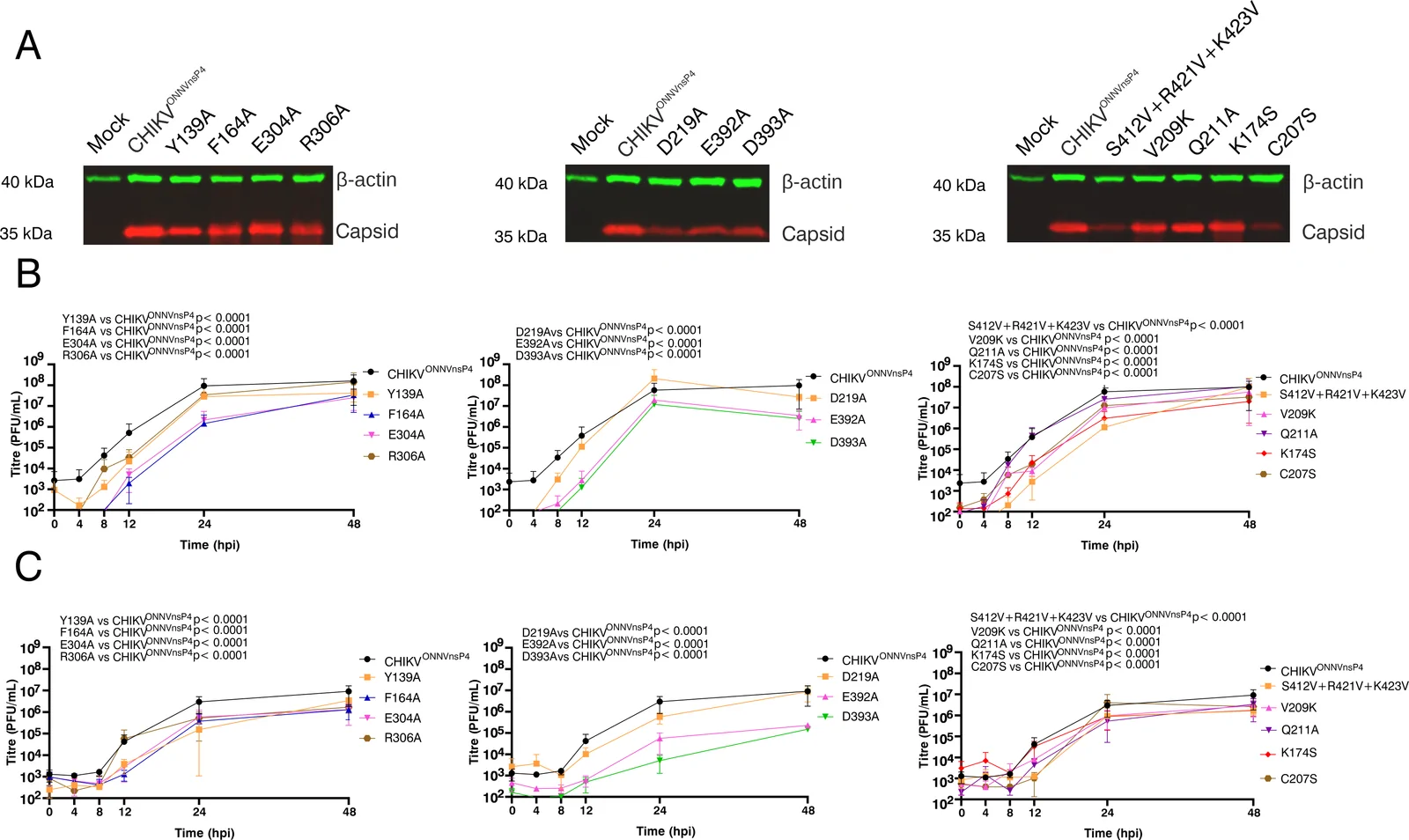
Mutations introduced into nsP4 of CHIKV^ONNVnsP4^ affect virus rescue and attenuate replication in mammalian and mosquito cells. (**A**) BHK-21 cells were transfected with plasmids containing icDNAs of parental CHIKV^ONNVnsP4^ or its variants harboring the indicated mutations. Cells were harvested upon the appearance of CPE or at 72 hpt. Cell lysates were analyzed by western blotting using antibodies against CHIKV capsid protein, with β-actin serving as a loading control. Representative results from one of three independent and reproducible experiments are shown. (**B and C**) BHK-21 cells (**B**) and *Aedes albopictus* C6/36 cells (**C**) were infected at a multiplicity of infection (MOI) of 0.01 with CHIKV^ONNVnsP4^ or its viable mutant variants harboring defects in nsP4–nsP1 (left panels), nsP4–nsP2 (middle panels), or putative nsP4–RNA (right panels) interactions. At the indicated time points, supernatants were collected, and virus titers were determined using a focus-forming assay. Data represent mean values ± SD from three independent experiments. Statistical significance of differences between the entire growth curves of mutant viruses and the parental virus was assessed using repeated-measures ANOVA; corresponding *P*-values are shown on each panel.

The infectious center assay (ICA) demonstrated that icDNA clones harboring mutations affecting nsP1–nsP4 or nsP2–nsP4 interactions retained high infectivity. Consistent with this, all rescued viruses replicated to high titers, although their peak titers were generally lower than those of the parental CHIKV^ONNVnsP4^ (Table S3). Successful replication of each mutant was further supported by the detection of capsid protein expression in transfected cells (Fig. 4A). Virus stocks were subsequently used to infect BHK-21 cells. All four viruses carrying mutations in nsP1-interacting residues exhibited delayed growth, with the most pronounced differences relative to parental CHIKV^ONNVnsP4^ observed at 12 h post-infection (hpi). While the R306A mutant approached parental titers at later time points, Y139A showed moderate attenuation, and the remaining two mutants (F164A and E304A) displayed more substantial attenuation throughout the course of infection. For viruses bearing mutations in nsP2-interacting residues, D219A showed clear attenuation at early time points but ultimately reached final titers comparable to parental CHIKV^ONNVnsP4^. In contrast, E392A and D393A exhibited more pronounced attenuation, with peak titers approximately 10-fold lower than those of CHIKV^ONNVnsP4^ (Fig. 4B). These observations are largely consistent with the *trans*-replication data, in which F164A and D393A caused strong attenuation. The contrasting behavior of R306A—strong attenuation in the *trans*-replication assay (Fig. 2) but improved growth at later time points (Fig. 4B)—suggests that adaptive mutations may have arisen during viral propagation. In *Aedes albopictus* C6/36 cells, viruses harboring mutations in nsP1–nsP4 interaction sites displayed characteristics broadly consistent with those observed in BHK-21 cells, with the notable exception of R306A. Unlike in BHK-21 cells, R306A remained attenuated at later time points in C6/36 cells, reaching titers similar to those of the other mutants (Fig. 4C). This behavior again suggests the possibility of adaptive mutations that enhance replication in mammalian cells but not in mosquito-derived cells. Similarly, all mutants affecting nsP2–nsP4 interactions exhibited growth attenuation comparable to that observed in BHK-21 cells, although the reduction in titers was somewhat more pronounced in C6/36 cells (Fig. 4C). Taken together, these data indicate that mutations in conserved nsP1–nsP4 and nsP2–nsP4 interaction residues have broadly similar effects in both cell types.

Of the nine chimeric viruses harboring mutations targeting putative RNA-interaction sites, five (C207S, K168S + R170S + K174S, C207A + V209K + Q211A, K264A, and H327A) produced no plaques in the ICA (Table S3), consistent with the strong attenuation of RNA replication observed in the *trans*-replicase assay (Fig. 3). Four of these mutants failed to produce infectious progeny; however, the virus carrying the C207S substitution replicated to reasonably high titers (Table S3), and weak but detectable capsid protein expression was observed by immunoblotting (Fig. 4D). This behavior strongly suggests that reversions, pseudoreversions, or second-site adaptive mutations occurred during rescue or propagation. The remaining four viruses produced plaques in the ICA, although plaque numbers were substantially reduced for the mutant containing the S412V + R421V + K423V triple substitution (Table S3). Consistently, this mutant virus reached the lowest final titers and displayed reduced capsid expression (Fig. 4D), in agreement with the decreased activity observed for the corresponding *trans*-replicase counterpart (Fig. 3). Reduced plaque numbers were also noted for the V209K mutant, which likewise exhibited reduced final titers (Table S3). The other two viruses—harboring Q211A or K174S mutations, both of which caused relatively mild attenuation in the *trans*-replicase assay (Fig. 3)—displayed plaque numbers comparable to the parental virus, although their titers were modestly reduced (Table S3). Both expressed capsid protein at levels similar to parental CHIKV^ONNVnsP4^ (Fig. 4D).

Growth kinetics in BHK-21 cells showed that the Q211A mutant was nearly indistinguishable from the parental chimeric virus. Somewhat unexpectedly, the K174S mutant exhibited clear and persistent attenuation at all time points examined. The triple mutant S412V + R421V + K423V was strongly attenuated early in infection but ultimately reached parental virus-like final titers. A similar, although less pronounced, delay was observed for the V209K mutant. The C207S virus—which likely depended on adaptive changes for replication—reached somewhat lower final titers (Fig. 4B). In C6/36 cells, the growth profiles of the mutant viruses were largely consistent with those observed in BHK-21 cells. The virus harboring K174S showed the lowest final titers, along with the mutant carrying the S412V + R421V + K423V triple substitution, which—unlike in BHK-21 cells—remained attenuated even at later time points (Fig. 4C).

In conclusion, of the 16 mutants tested in the context of the CHIKV^ONNVnsP4^, 12 were successfully rescued. Overall, the results showed good concordance with those obtained in the *trans*-replicase assay. Some viruses harboring mutations in the structurally defined nsP1–nsP4 or putative RNA–nsP4 interaction sites displayed evidence of adaptation in mammalian cells but remained attenuated in mosquito cells.

### Impact of nsP4 mutations affecting interactions within replicase core on SFV is similar to their effects in the CHIKV^ONNVnsP4^ chimera

Experiments using the CHIKV^ONNVnsP4^ chimera indicated that the negative effects of several nsP4 mutations were frequently mitigated by reversions, pseudoreversions, or second-site compensatory mutations. Although the analysis of such adaptive changes is technically feasible in the chimeric CHIKV^ONNVnsP4^ system, interpretation is complicated by the artificial genomic context—specifically, the presence of ONNV nsP4 within a CHIKV backbone. To address this limitation and validate the attenuation phenotypes in a more natural setting, we extended our analysis to SFV, a well-established alphavirus model that has been instrumental in elucidating the mechanisms of alphavirus genome replication, including the role of nsP4 in RNA synthesis. SFV also replicates to very high titers, facilitating the detection of replication-competent but strongly attenuated mutants that might otherwise be misclassified as non-infectious. Importantly, all nsP4 residues analyzed in the context of CHIKV^ONNVnsP4^ are identical in ONNV and SFV (Fig. 1B), allowing direct introduction of the same substitutions into the genome of the SFV6 isolate, selected for its well-characterized virulent phenotype in mice.

**Fig 5:**
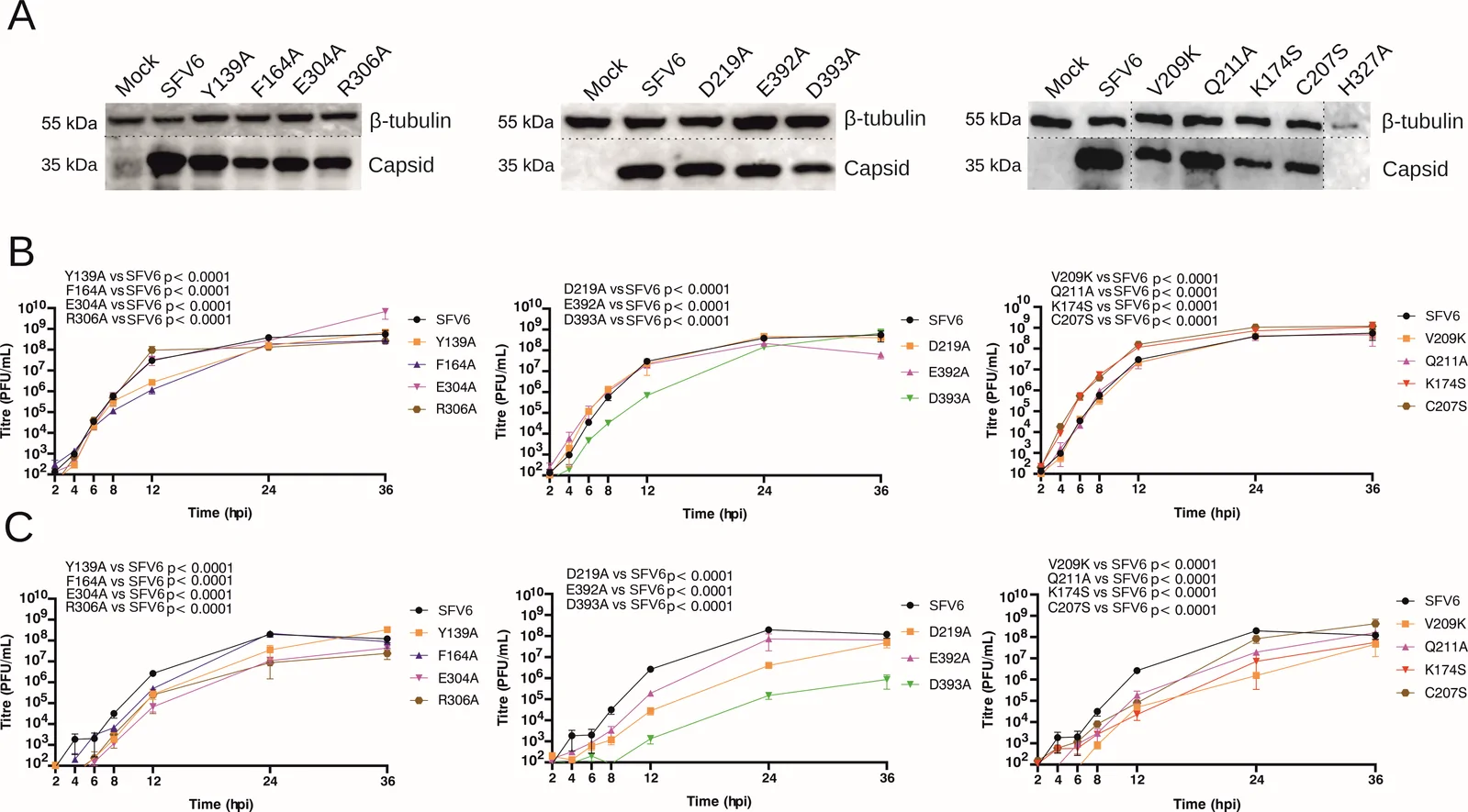
Mutations introduced into nsP4 of SFV6 affect virus rescue and attenuate replication. (**A**) BHK-21 cells were transfected with plasmids containing infectious cDNAs of wt SFV6 or its variants harboring the indicated mutations. Cells were harvested upon the appearance of CPE or at 72 hpt. Cell lysates were analyzed by western blotting using antibodies against SFV capsid protein, with β-tubulin serving as a loading control. Representative results from one of three independent and reproducible experiments are shown. Places where original images were cut are represented with dotted lines. (**B and C**) BHK-21 cells (**B**) and *Aedes albopictus* C6/36 cells (**C**) were infected at an MOI of 0.01 with wt SFV6 or its viable mutant variants harboring defects in nsP4–nsP1 (left panels), nsP4–nsP2 (middle panels), or putative nsP4–RNA (right panels) interactions. The experiment was performed, and data are shown as described for Fig. 4B and C, except that the virus titers were determined using a plaque assay.

All SFV genomes harboring mutations targeting nsP1-interaction sites were successfully rescued, replicated to high titers (Table S3), and expressed capsid protein levels that were only slightly reduced relative to the wt virus (Fig. 5A). Consistent with these observations, all mutants exhibited attenuation in BHK-21 cells. However, SFV variants carrying the E304A or R306A substitutions displayed growth kinetics comparable to wt SFV6, whereas viruses harboring Y139A or F164A showed more pronounced reductions in replication, particularly at early time points (Fig. 5B). These phenotypes closely mirrored those observed for the corresponding mutations in the CHIKV^ONNVnsP4^ chimera, consistent with the high conservation of the targeted nsP4 residues among alphaviruses.

Similarly, all SFV variants containing mutations in nsP2-interaction sites exhibited some degree of attenuation (Table S3; Fig. 5A). Growth-curve analysis in BHK-21 cells revealed that the D393A mutant was the most strongly attenuated, particularly at early time points (Fig. 5B). The virus harboring the E392A substitution showed milder but persistent attenuation, most evident at later time points, whereas the D219A mutant displayed growth kinetics closely resembling those of wt SFV6 (Fig. 5B). Growth-curve analysis in C6/36 cells showed that viruses with mutations in nsP4–nsP1 interaction sites largely retained phenotypes similar to those observed in mammalian cells, except that attenuation of E304A- and R306A-bearing viruses was more pronounced. A similar trend was observed for all three mutants affecting the nsP4–nsP2 interface, which showed phenotypes comparable to those in BHK-21 cells but with enhanced attenuation in mosquito cells (Fig. 5C). Overall, the similarity of growth profiles in mammalian and mosquito cells suggests that the functional consequences of altering conserved nsP4 residues involved in interactions with other replicase subunits are broadly conserved across cell types.

In contrast, mutations targeting putative RNA-interaction sites of nsP4 produced more divergent phenotypes in SFV compared to those observed in the CHIKV^ONNVnsP4^ chimera. Most notably, the SFV mutant carrying the H327A substitution was viable—albeit replicating to very low titers and producing no detectable capsid protein by immunoblotting—whereas the SFV variant harboring the S412V + R421V + K423V triple substitution was non-viable (Table S3). These differences may reflect intrinsic distinctions between the viral systems used. Consistent with results obtained using the CHIKV^ONNVnsP4^ chimera, the K168S + R170S + K174S, C207A + V209K + Q211A and K264A substitutions were lethal for SFV. As observed in the chimeric background, the SFV mutant carrying the C207S substitution failed to form plaques in the ICA assay but nonetheless replicated efficiently and expressed capsid protein (Table S3; Fig. 5A), again suggesting the emergence of adaptive mutations.

SFV variants harboring the V209K, Q211A, or K174S substitutions were successfully rescued; among these, the K174S mutant exhibited reduced titers and the lowest capsid protein levels (Table S3; Fig. 5A). However, growth-kinetics analysis in BHK-21 cells revealed that not only the Q211A mutant—which reached the final titers comparable to wt SFV6 and expressed the highest capsid protein levels—but also all other rescued SFV variants with mutations in putative RNA-interaction sites (with the exception of H327A, which was excluded due to extremely low titers) replicated to levels nearly indistinguishable from wt SFV6 and no longer displayed pronounced attenuation (Fig. 5B). This behavior contrasts with that observed in the CHIKV^ONNVnsP4^ chimera (Fig. 4B); most notably, the K174S substitution, which caused strong attenuation in the chimera, had minimal impact on SFV replication in BHK-21 cells.

Thus, unlike mutations affecting nsP4 interactions with nsP1 or nsP2—where phenotypes were largely conserved across both viral systems—the effects of mutations in putative RNA-interacting residues differed substantially. This divergence suggests either that the functional roles of these residues differ between SFV and CHIKV^ONNVnsP4^ or, more likely, that in the context of a natural virus, such as SFV, deleterious effects of these mutations are efficiently compensated by reversions, pseudoreversions, or second-site mutations arising during rescue and propagation in mammalian cells. Growth-kinetics experiments in C6/36 mosquito cells support the latter interpretation: in contrast to mammalian cells, all analyzed mutants exhibited clear attenuation—except at the final 36 hpi time point—with one of the most pronounced effects observed for the virus carrying the K174S substitution (Fig. 5C).

### SFV genomes with mutations affecting putative nsP4–RNA interaction residues frequently increase replication fitness through pseudoreversion and/or second-site compensatory mutations

To investigate the molecular basis of the increased replication fitness observed for SFV genomes carrying mutations in nsP4, we plaque-purified six isolates from each rescued virus stock, amplified the region encoding the ns proteins by RT-PCR, and subjected the resulting fragments to Sanger sequencing. None of the analyzed viruses exhibited reversion of the engineered mutations; however, pseudoreversions and/or second-site compensatory mutations were detected in several variants (Table S4).

Among SFV genomes harboring substitutions in nsP1–nsP4 interaction sites, no potential adaptive changes were identified in viruses containing the Y139A, F164A, or E304A mutations. The R306A mutant was likewise largely stable: four of six analyzed isolates contained no additional changes, while the remaining two carried distinct second-site mutations, F191L in nsP4 or R623K in nsP2 (Table S4). Given the high rescue efficiency of this mutant (Table S3) and the predominance of clones lacking adaptive substitutions, these changes—if adaptive at all—are unlikely to have contributed substantially to viral replication in BHK-21 cells (Fig. 5B). A similar pattern was observed for mutants affecting the nsP2–nsP4 interface. No compensatory mutations were detected for viruses harboring D219A or E392A, and only one of six D393A-derived isolates contained a second-site substitution in nsP3 (Table S4). Collectively, these data indicate that mutations introduced into conserved nsP4 residues involved in interactions with other replicase core subunits are stably maintained, and viruses carrying disadvantageous changes remain attenuated (Fig. 5B) with little evidence of fitness restoration through adaptation.

In contrast, viruses carrying mutations predicted to disrupt nsP4–RNA interactions exhibited markedly different behavior. Of the five viable RNA-interaction mutants, only the Q211A virus showed minimal signs of adaptation; no changes were detected in four of the six analyzed clones. The remaining two clones contained distinct substitutions in nsP3 (Table S4), changes that likely represent random variation rather than targeted adaptation. Consistently, analysis of progeny of the CHIKV^ONNVnsP4^ chimera harboring the Q211A mutation also revealed no reversions, pseudoreversions, or adaptive substitutions. The other four mutants, however, exhibited clear and consistent evidence of adaptation, albeit adaptation patterns varied between mutants. All six isolates of the C207S virus contained the same pseudoreversion, in which the engineered Ser was replaced by Phe. The absence of additional mutations indicates that this pseudoreversion was both necessary and sufficient to enable virus rescue compromised by the original mutation (Table S3). The CHIKV^ONNVnsP4^ chimeric virus carrying the C207S substitution—which exhibited a similar defect in rescue efficiency—also displayed genetic instability. Interestingly, none of the analyzed isolates of this virus contained the above-described pseudoreversion; instead, two isolates harbored an A161T substitution, and four isolates harbored an A202D substitution in nsP4. SFV containing the K174S substitution also acquired compensatory changes, but with greater diversity. Four isolates harbored an H176R substitution in nsP4, one contained an E316K substitution, and one isolate carried both changes. Notably, both substitutions introduce a positively charged residue (Arg or Lys), potentially compensating for the loss of positive charge caused by the original K174S mutation. In contrast to SFV, and consistent with the persistent attenuation of this virus in BHK-21 cells (Fig. 4B), no adaptive changes were detected for the CHIKV^ONNVnsP4^ chimeric virus carrying the K174S substitution. For SFV harboring the V209K mutation, one isolate lacked adaptive changes, and another contained an E399G substitution in nsP2, a change of uncertain relevance. The remaining four clones carried heterogeneous but potentially adaptive substitutions, including replacement of the acidic Glu residue at position 481 in nsP4 with Lys (E481K), alone or in combination with a pseudoreversion that introduced a Thr residue at position 209. Another pseudoreversion, introducing a hydrophobic Ile residue at position 209, was found in combination with additional substitutions (S183N and D390E) in nsP3 (Table S4). Finally, adaptation analysis of the severely attenuated SFV H327A mutant revealed a predominant second-site mutation, Q192R in nsP4, present in four of six isolates, while the remaining two clones contained either V187I alone or V187I in combination with S422I in nsP3.

Taken together, these results reveal multiple adaptive pathways by which SFV can compensate for defects in putative RNA-interacting residues of nsP4. The functional significance of these adaptive changes and the molecular mechanisms by which they restore replicase activity warrant further investigation. In contrast, the CHIKV^ONNVnsP4^ chimera displayed a somewhat reduced ability to acquire adaptive mutations, and when such changes were detected, they differed from those observed in SFV.

### F164A, K174S, and D393A substitutions attenuate CHIKV replication *in vitro*

Although identical mutations introduced into conserved positions of nsP4 in CHIKV^ONNVnsP4^ and SFV6 produced broadly similar effects on viral replication *in vitro*, the resulting phenotypes were not identical (Table S3; Fig. 4 and 5). Furthermore, the viruses differed in the genetic stability of mutant variants and in the mechanisms used to compensate for the negative effects of the introduced mutations. To extend this analysis, we selected the F164A, K174S, and D393A substitutions—each associated with a clear attenuated phenotype and affecting interactions with nsP1, RNA, and nsP2, respectively—and introduced them into an authentic CHIKV backbone.

**Fig 6:**
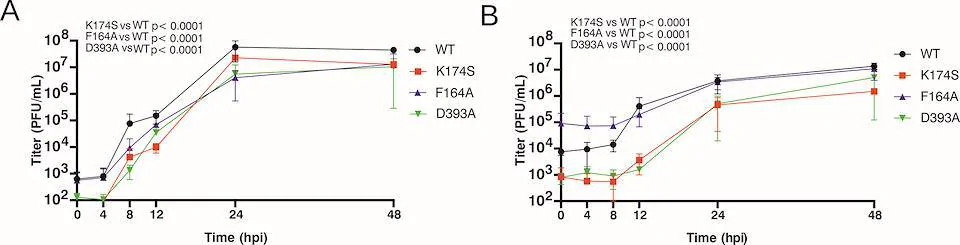
Mutations in nsP4 affecting interactions with nsP1, nsP2, or RNA attenuate CHIKV *in vitro*. BHK-21 cells (**A**) and *Aedes albopictus* C6/36 cells (**B**) were infected at an MOI of 0.01 with wt CHIKV, CHIKV^F164A^, CHIKV^K174S^, and CHIKV^D393A^. The experiment was performed, and data were analyzed as described for Fig. 4B and C.

ICA revealed that CHIKV harboring any of the three mutations exhibited reduced rescue efficiency, with decreases ranging from ~9-fold for CHIKV^F164A^ to nearly 100-fold for CHIKV^K174S^. Growth-kinetic analysis in BHK-21 cells showed that all three mutants were significantly attenuated relative to wt CHIKV. Attenuation was more pronounced for CHIKV^F164A^ and CHIKV^D393A^, whereas CHIKV^K174S^ reached titers comparable to those of wt CHIKV by 24 hpi (Fig. 6A), likely reflecting the accumulation of adaptive mutations. Indeed, analysis of the rescued CHIKV^K174S^ stock revealed the H176R substitution, previously observed in SFV^K174S^ (Table S4), in two of the six sequenced clones. Thus, with respect to compensatory changes, CHIKV^K174S^ more closely resembles SFV^K174S^ than its chimeric CHIKV^ONNVnsP4^ counterpart. Consistent with this interpretation, CHIKV^K174S^ displayed the strongest attenuation in C6/36 cells, with titers at least 10-fold lower than those of wt CHIKV at every time point. CHIKV^D393A^ showed a similar degree of attenuation, whereas interestingly, CHIKV^F164A^ replicated efficiently in insect cells (Fig. 6B), a property consistent with that observed for SFV carrying the same substitution (Fig. 5C).

### Mutations affecting nsP4 interactions with other replicase subunits strongly attenuate SFV *in vivo*

Non-attenuated SFV6 is neurovirulent in adult mice, causing high viremia and fatal encephalitis, and was therefore used to evaluate the *in vivo* effects of nsP4 mutations associated with distinct *in vitro* phenotypes. SFV^F164A^ and SFV^D393A^ were attenuated and exhibited high genetic stability, whereas the virus stock rescued from the icDNA of SFV^K174S^ consisted of a mixture of at least three variants carrying potential adaptive mutations (Table S4). This stock showed replication kinetics comparable to those of wt SFV in BHK-21 cells but was strongly attenuated in mosquito-derived cells (Fig. 5B and C).

**Fig 7:**
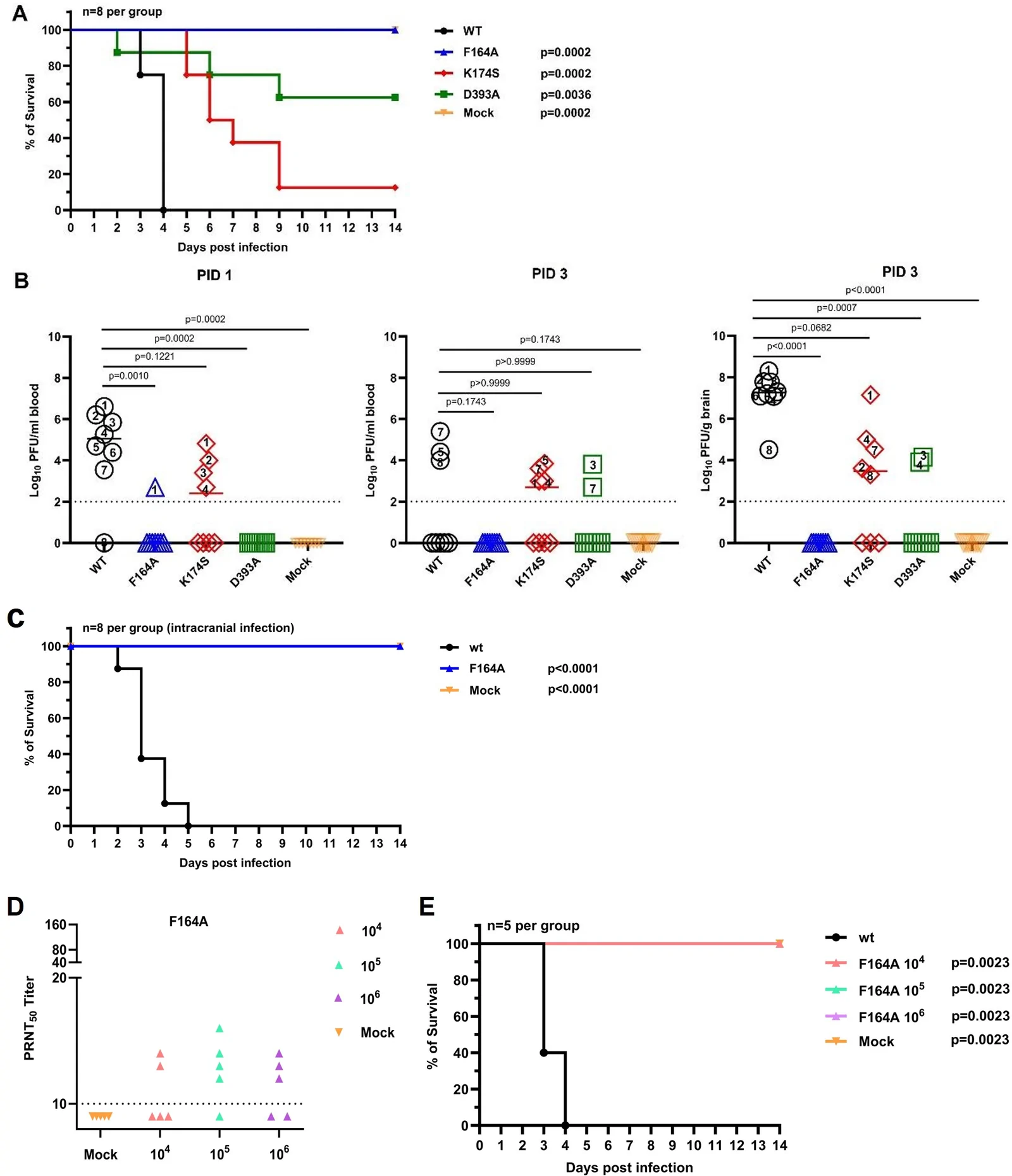
Mutations in nsP4 affecting interactions with nsP1, nsP2, or RNA attenuate SFV *in vivo*. (**A**) Six-week-old Kunming mice were inoculated i.p. with 1,000 PFU of wt SFV6, SFV^F164A^, SFV^K174S^, or SFV^D393A^, or mock infected (*n* = 8 per group) and monitored for up to 14 days for clinical signs. Statistical significance between wt SFV-infected and other groups, analyzed using the log-rank (Mantel–Cox) test, is shown. (**B**) Six-week-old Kunming mice (*n* = 8 per group) were infected as described in panel A. Blood samples were collected on PID 1 and 3, after which the animals were euthanized, and brains were harvested for the determination of viral titers by plaque assay. Data points represent individual animals; each animal within an infection group is assigned a unique identifier shown in the figure. Statistical significance between wt SFV-infected and other groups was analyzed using the Kruskal-Wallis test. (**C**) Six-week-old Kunming mice (*n* = 8 per group) were inoculated intracranially with 1,000 PFU of wt SFV6, SFV^F164A^, or mock-infected and monitored for up to 14 days for clinical signs. (**D**) Six-week-old Kunming mice were immunized i.p. with 10⁴, 10⁵, or 10⁶ PFU of SFV^F164A^, or mock-immunized (*n* = 5 per group), and the titers of neutralizing antibodies at 2 weeks post-immunization were determined using plaque reduction neutralization tests (PRNTs). The dotted line (**B and D**) indicates the limit of detection. (**E**) Four-week-old Kunming mice were immunized i.p. with 10⁴, 10⁵, or 10⁶ PFU of SFV^F164A^ (*n* = 5 per group) or mock-immunized (*n* = 10). Two weeks later, the mice were challenged i.p. with 1,000 PFU of wt SFV6. Of the mock-immunized mice, five were challenged with wt SFV6 (wt), and five were mock-infected (mock). Animals were monitored for up to 14 days for clinical signs. Statistical significance between groups (**A, C, and E**) was analyzed using the log-rank (Mantel-Cox) test.

In the first experiment, groups of 6-week-old Kunming mice (*n* = 8 per group) were intraperitoneally (i.p.) inoculated with 1,000 PFU of each mutant or wt SFV and monitored for 14 days for clinical signs. As expected, all mice infected with wt SFV developed severe symptoms and were euthanized on post-infection day (PID) 3 or 4, whereas mock-infected animals remained healthy (Fig. 7A). Seven of eight animals infected with SFV^K174S^ succumbed between PID 5 and 9, indicating that at least one variant present within the mixed virus stock retained sufficient virulence—consistent with its efficient replication in mammalian cells (Fig. 5B). One mouse infected with SFV^D393A^ died at PID 2 from causes unrelated to infection. Among the remaining seven animals, one died at PID 6 and one at PID 9, whereas five survived until the end of the monitoring period. In contrast, none of the mice infected with SFV^F164A^ exhibited any clinical signs, and all survived through the full observation period. Thus, both SFV^D393A^ and SFV^F164A^ display strong attenuation *in vivo* (Fig. 7A), with the difference between their phenotypes likely reflecting the ability of SFV^D393A^ to eventually reach titers comparable to wt SFV in cell culture, in contrast to the persistently reduced replication of SFV^F164A^ (Fig. 5B).

In a second experiment, designed to dissect the basis of the distinct phenotypes exhibited by the analyzed viruses, groups of mice (*n* = 8 per group) were infected as described above. Blood samples were collected on PID 1 and PID 3; after the second sampling, animals were euthanized, and their brains were harvested for the determination of viral titers. At PID 1—typically corresponding to the peak of wt SFV viremia—seven of eight wt SFV-infected mice displayed detectable viremia. Viremia was likewise detected in four mice infected with SFV^K174S^, although titers were lower than those in wt SFV-infected animals. Consistent with the avirulent phenotype observed in the survival experiment, only one SFV^F164A^-infected mouse had detectable viremia, and none of the attenuated SFV^D393A^-infected mice tested positive (Fig. 7B, left panel). At PID 3, viremia persisted only in three wt SFV-infected mice—including one animal that had tested negative on PID 1 (Fig. 7B, middle panel). Virus was nevertheless detected in the brains of all eight wt SFV-infected animals, demonstrating efficient neuroinvasion (Fig. 7B, right panel). Among SFV^K174S^-infected mice, viremia was again detected in four animals—including two that were negative on PID 1—and virus was present in the brains of five mice, although titers were substantially lower than those in wt SFV-infected animals (Fig. 7B, middle and right panels). Two mice in the SFV^D393A^ group had detectable viremia, and two harbored virus in the brain (Fig. 7B). As expected from its avirulent phenotype, no SFV^F164A^-infected animals had detectable virus in the serum or brain at PID 3. To determine whether the absence of disease resulted solely from impaired neuroinvasion or also reflected reduced neurovirulence, mice were inoculated intracranially with SFV^F164A^. All inoculated animals survived, confirming the strongly attenuated phenotype of the mutant virus (Fig. 7C). Taken together, these findings show that the attenuated phenotypes of the SFV^K174S^ and SFV^D393A^ correlate with strongly reduced or absent viremia, indicating that diminished peripheral replication limits efficient neuroinvasion, thereby reducing pathogenicity. Once the virus enters the brain, however, disease progresses to fatal encephalitis. This conclusion is supported by analysis of brain tissues from the first experiment, in which animals reaching clinical endpoints harbored very high viral loads in the brain (>10¹⁰ PFU/g for wt SFV and ~10⁸ PFU/g for SFV^K174S^- or SFV^D393A^-infected mice). In contrast, the avirulent phenotype of SFV^F164A^ appears to result not only from impaired peripheral replication but also from a lack of neurovirulence.

Finally, we tested whether infection with avirulent SFV^F164A^ induces neutralizing antibodies and protective immunity. In the first experiment, 6-week-old mice (*n* = 5 per group) were immunized with 10⁴, 10⁵, or 10⁶ PFU of SFV^F164A^, or mock-immunized, and monitored for disease signs for 14 days. Regardless of the dose used, all immunized animals survived without displaying any clinical signs. Serum samples collected at PID 14 were analyzed by PRNTs. Approximately 50% of immunized mice had PRNT_50_ titers below the detection limit, whereas the remaining animals exhibited low-level responses. No correlation with the administered infectious dose was observed, suggesting that immunity was primarily induced by viral progeny rather than by virus present in the inoculum (Fig. 7D). In a second experiment, 4-week-old mice were immunized with 10⁴, 10⁵, or 10⁶ PFU of SFV^F164A^ or mock-immunized. Two weeks later (i.e., when animals reached 6 weeks of age), they were challenged with a lethal dose (1,000 PFU) of wt SFV6. All mock-immunized animals challenged with SFV6 succumbed to infection by 4 days post-challenge, whereas all immunized animals survived (Fig. 7E). Thus, immunization with SFV^F164A^ provided complete protection against lethal SFV challenge, even at the lowest immunization dose.

### F164A, K174S, and D393A substitutions attenuate CHIKV-induced disease in mice

**Fig 8:**
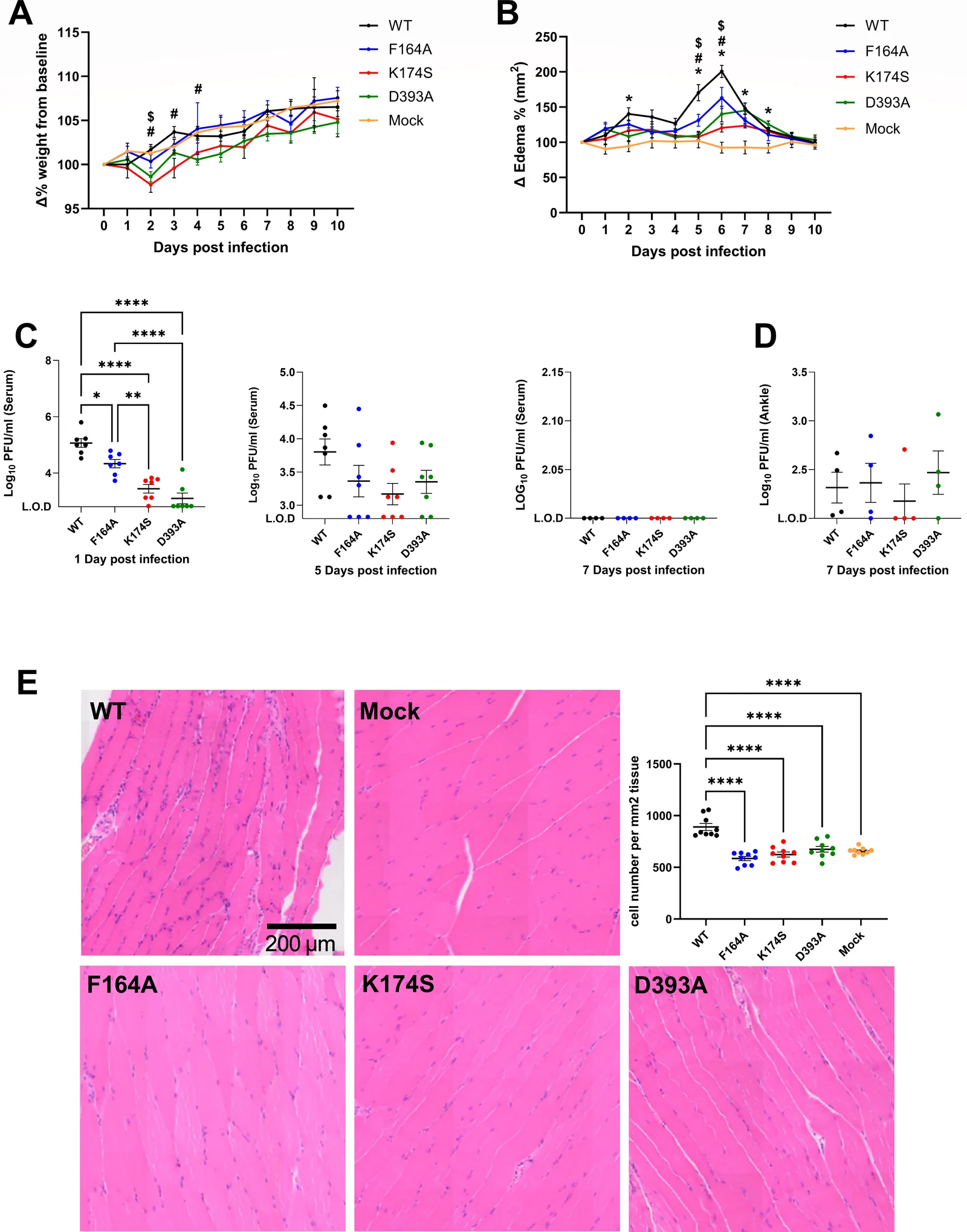
CHIKV-induced joint inflammation is reduced in mice infected using CHIKV^F164A^, CHIKV^K174S^, and CHIKV^D393A^. (**A–C**) Adult C57BL/6J mice were inoculated subcutaneously in the footpad with wt CHIKV, CHIKV^F164A^, CHIKV^K174S^, and CHIKV^D393A^ or were mock-infected with PBS (*n* = 7 mice per group for panels A and B. For panel C, *n* = 7 mice per group for days 1 and 5 serum samples, and *n* = 4 mice per group for day 7 serum samples). (**A**) Body weight gain and (**B**) foot swelling, expressed as the change in foot height × width relative to day 0, were measured. Statistical differences between groups at each time point were assessed using two-way ANOVA. Symbols indicate significant differences as follows: *, wt CHIKV versus PBS; #, wt CHIKV versus CHIKV^D393A^; $, wt CHIKV versus CHIKV^K174S^. (**C**) Viremia in the serum was determined by plaque assay. Statistical differences between groups were assessed using one-way ANOVA. *, *P*  <  0.05; **, *P*  <  0.01; ****, *P*  <  0.0001. (**D and E**). Adult C57BL/6J mice were inoculated subcutaneously in the footpad with wt CHIKV, CHIKV^F164A^, CHIKV^K174S^, and CHIKV^D393A^ or were mock-infected with PBS (*n* = 4 per group). Animals were euthanized at PID 7. (**D**) Viral titers in ankle tissues were determined by plaque assay. Ankle tissues were homogenized in a fixed volume of 1 mL PBS, independent of tissue weight. PFU values were then normalized to tissue mass by applying a correction factor based on the ratio between the heaviest sample and each individual sample. (**E**) Hematoxylin and eosin (H&E) staining was performed on quadriceps sections collected at PID 7. Nuclear staining was quantified using QuPath. Statistical differences between groups were assessed using one-way ANOVA. ****, *P*  <  0.0001. Each dot represents one section.

To determine whether the attenuated *in vitro* phenotypes of CHIKV^F164A^, CHIKV^K174S^, and CHIKV^D393A^ were also associated with reduced disease *in vivo*, adult C57BL/6J mice were inoculated subcutaneously in the footpad with wt CHIKV or the indicated mutant viruses and monitored for 10 days. Mouse body weight gain was comparable between virus-infected and mock-infected animals (Fig. 8A). Foot swelling was first observed in wt CHIKV-infected mice at PID 2, peaked at PID 6, and resolved by PID 10 (Fig. 8B). All three mutant viruses induced significantly lower disease scores than wt CHIKV throughout the period of disease symptoms (Fig. 8B). No significant differences were detected among mice infected with CHIKV^F164A^, CHIKV^K174S^, or CHIKV^D393A^, although disease severity tended to be greater in CHIKV^F164A^-infected animals.

To determine whether reduced disease was associated with altered viral replication, serum samples were collected at PID 1, 5, and 7 for plaque assay. At PID 1, wt CHIKV produced significantly higher serum titers than any of the three mutant viruses (Fig. 8C). Among the mutants, CHIKV^F164A^ produced significantly higher serum titers than CHIKV^K174S^ and CHIKV^D393A^, consistent with its less attenuated phenotype *in vivo* and with the higher viral titers observed in BHK-21 cells at early time points (Fig. 6A). By PID 5, serum titers did not differ significantly among groups, and infectious virus was undetectable in serum by PID 7 (Fig. 8C).

In another experiment, ankle joints and quadriceps were collected at PID 7. No significant differences in viral titers in ankle joints were observed among the groups (Fig. 8D). H&E-stained quadriceps sections collected on PID 7 showed greater inflammatory cell infiltration and tissue pathology in wt CHIKV-infected mice than in mice infected with any of the three mutant viruses (Fig. 8E). Consistent with the disease scores, no obvious differences in inflammatory infiltrates were observed among the three mutant groups. Together, these findings demonstrate that the F164A, K174S, and D393A substitutions attenuate CHIKV replication and CHIKV-induced musculoskeletal disease *in vivo*.

## DISCUSSION

nsP4 is the most conserved nonstructural protein of alphaviruses and the key enzyme of viral RNA replication, making it an attractive target for antiviral intervention. Consistent with this, several recently described antiviral compounds target nsP4, either as substrate mimetics or as allosteric inhibitors (43, 49). Despite its central role, however, our understanding of nsP4 function remains incomplete. Mutational analyses have been instrumental in defining its roles during infection. A previous study focusing on the NTD of nsP4 analyzed 29 mutant genomes and revealed the effects on both negative-strand and subgenomic RNA synthesis, as well as compensatory mutations in nsP1, nsP2, and nsP3, suggesting that the NTD interacts with other nonstructural proteins in functionally distinct replication complexes. The same study also indicated a possible role for nsP4 in the inhibition of host protein synthesis (50). Structure-guided mutagenesis has further identified functionally critical regions of nsP4, including segments appearing disordered in crystal structures (46), while modeling-based analyses suggested that distinct nsP4 subdomains contribute not only to RNA synthesis but also to efficient virion production (51). Collectively, these studies highlight the multifunctional nature of nsP4. Here, we extend these analyses by targeting regions of nsP4 involved in interactions with nsP1, nsP2, or viral RNA. We assessed 58 mutations in a *trans*-replicase system and further characterized selected variants in chimeric and authentic viral systems (CHIKV^ONNVnsP4^, SFV, and CHIKV), as well as in SFV and CHIKV mouse models.

Mutations targeting nsP4 interactions with nsP1 or nsP2 often drastically reduced *trans*-replicase activity, most likely by disrupting conserved structural interfaces within the replicase complex. In the context of viral genomes, mutations with partial effects on RNA replication nevertheless resulted in clear attenuation of viral growth across all systems analyzed. These mutant viruses displayed broadly similar attenuated phenotypes in both mammalian and mosquito cells, with the notable exception of R306A, which showed evidence of adaptation in mammalian cells. Consistent with this, no prominent secondary mutations in nsP4 or other nonstructural proteins were detected. The apparent discrepancy from earlier studies reporting compensatory mutations likely reflects the selection of variants with relatively mild defects in rescue and propagation (Table S3), which may have limited the selective pressure for rapid adaptation.

Mutations in residues lining the putative RNA channel, guiding interface, RNA entry site, and other predicted RNA-interacting regions further support the functional importance of these elements. One notable exception is the CTD tail (residues 608–611), which does not appear to be required for RNA priming or initiation, in contrast to the CTD of the flaviviral polymerase NS5 (52). In contrast, the NTD of nsP4 is clearly critical and may contribute to RNA coordination during initiation and elongation. Consistent with previous findings (50), deletions and substitutions in this region (e.g., del1–15, del9–10, and 58–73 substitutions) nearly abolished replicase activity. Our data further suggest that specific NTD residues may contribute to interactions with nsP1 and, potentially more prominently, with viral RNA. Although the precise mechanism remains to be established, the first 15 amino acids of the NTD may influence access to the RNA-binding tunnel leading to the GDD active site, whereas residues 53–73 and K264 may contribute to RNA positioning or stabilization during RNA synthesis. Additional residues in the palm domain may influence RNA-dependent polymerase activity or NTP incorporation. Direct biochemical studies will be required to define these roles mechanistically. Consistent with these proposed roles, mutations in these regions abolished or strongly attenuated replicase activity and viral replication.

Consistent with these observations, adaptive changes were primarily associated with mutations affecting predicted nsP4–RNA interactions and predominantly mapped to nsP4. These adaptations involved diverse mechanisms, including pseudoreversions (e.g., C207S→C207F in SFV^C207S^) and second-site substitutions that restored charge or structural stability (e.g., H176R in SFV^K174S^ and CHIKV^K174S^). Such changes improved viral fitness in mammalian cells but were often ineffective in mosquito cells, underscoring distinct functional constraints imposed on the replicase in vertebrate and invertebrate hosts. More broadly, this host-specific pattern of adaptation highlights the importance of evaluating attenuation strategies in both systems. In contrast to a previous study (50), mutations in other nonstructural proteins were rare and did not overlap with previously described compensatory changes; moreover, their functional relevance remains to be established. These differences are most likely attributable to the nature of the attenuating mutations, particularly their targeting of predicted nsP4–RNA interactions in the present study.

A notable observation is that nsP4 residue 192, previously linked to resistance to the ribonucleoside analog 4′-FlU (44), also emerged as a site of adaptive mutation. This suggests that residue 192 may represent a functional hotspot. Its location near predicted RNA-interacting regions supports the idea that substitutions at this site may modulate RNA binding and/or polymerase activity. Accordingly, adaptive mutations may restore replicase function, whereas resistance-associated substitutions may alter substrate usage or fidelity. More broadly, the overlap between adaptive and resistance-associated sites (including residue 483 (44, 53, 54)) suggests that certain nsP4 positions act as multifunctional regulatory nodes capable of tuning replicase activity under different selective pressures. This has implications for both alphavirus biology and antiviral development, including the potential for cross-resistance.

These findings also have implications for vaccine design. Despite recent advances, vaccines against medically important alphaviruses remain limited. Our results demonstrate that targeting conserved nsP4 residues provides a rational attenuation strategy. Because these residues are highly conserved, mutations disrupt core replication functions without altering structural proteins or antigenicity. Importantly, F164A, D393A, and K174S produced attenuation across three viral backbones, supporting the potential generalizability of this approach. However, validation in additional medically relevant alphaviruses will be required. The attenuation profile was supported by reduced replication, lower infectivity, and decreased viral titers *in vitro*. These effects translated *in vivo*, where attenuated mutants exhibited reduced viremia and limited neuroinvasion in the SFV model, as well as reduced serum viremia, musculoskeletal disease, and inflammatory cell infiltration in the CHIKV model. Notably, mutations affecting nsP4 interactions with nsP1 or nsP2 combined attenuation with high genetic stability, whereas mutations affecting predicted RNA interactions were more prone to adaptation, highlighting the importance of careful mutation selection.

Analysis of immune responses showed that animals vaccinated with SFV^F164A^ developed neutralizing antibodies, indicating that prominent attenuation did not abolish immunogenicity. Although PRNT titers were low, and in some cases below the level of detection, a separate group of immunized animals was fully protected against challenge with virulent SFV6. The combination of protective immunity with very limited replication represents a highly favorable outcome, minimizing both pathogenicity and the risk of mosquito-mediated dissemination. However, a number of important questions remain, including the duration of protection and the extent of cross-protection against heterologous alphaviruses. The CHIKV *in vivo* data also show that the relative effects of individual mutations are not identical across alphaviruses. In SFV, F164A produced the strongest attenuation, whereas in CHIKV, this mutant retained higher serum titers and a less attenuated disease phenotype than K174S or D393A. Thus, although conserved nsP4 residues provide promising targets for rational attenuation, the phenotypic impact of each mutation must be validated experimentally in each viral background.

In conclusion, this study demonstrates the power of structure-guided design for investigating alphavirus RNA replication and for developing rational attenuation strategies. Validation of mutation-associated phenotypes across multiple viral systems, combined with integration of *in vitro* and *in vivo* analyses, supports the feasibility of nsP4-based attenuation. Identification of adaptive pathways further provides insight into mechanisms influencing the stability of attenuated strains. Overall, targeting conserved nsP4 functions represents a promising approach for rational attenuation of alphaviruses. Future studies should focus on optimizing mutation combinations to balance attenuation, stability, and immunogenicity, and on evaluating their translational potential in advanced preclinical models.

## MATERIALS AND METHODS

### Structure-guided selection of interface mutations

Mutations in nsP4 were selected based on structural analysis of the CHIKV nsP1:nsP2:nsP4 replication complex (35, 46). Candidate residues were prioritized if they were located within 5 Å of nsP1 or nsP2, contributing to predicted hydrogen bonds, salt bridges, hydrophobic contacts, or buried surface area at the interface. To avoid disrupting global polymerase folding, substitutions were restricted to surface-exposed residues outside conserved catalytic motifs (A–F). Sequence conservation across representative alphaviruses was assessed to prioritize structurally and evolutionarily relevant positions.

### Cells

U2OS human osteosarcoma cells (ATCC HTB-96) were maintained in Iscove’s modified Eagle’s medium (IMDM; Corning) supplemented with 10% fetal bovine serum (FBS) and 2 mM L-glutamine at 37°C in a 5% CO_2_ atmosphere. BHK-21 cells (ATCC CCL-10) were cultured in Glasgow’s minimal essential medium (GMEM; Gibco) supplemented with 10% FBS and 200 mM HEPES (pH 7.2) at 37°C in 5% CO_2_. Vero E6 cells (ATCC CRL-1586) were cultured in Dulbecco’s modified Eagle medium (DMEM; Gibco) supplemented with 10% FBS at 37°C in 5% CO_2_. *A. albopictus* C6/36 cells were maintained in Leibovitz’s L-15 medium supplemented with 10% FBS and 10% tryptose phosphate broth (TPB) at 28°C without additional CO_2_.

### Plasmids

Human RNA polymerase I promoter-based expression plasmid designed to produce replication-competent template RNA of CHIKV (HSPolI-FG-CHIKV, where FG denotes Fluc and Gluc markers) as well as the expression plasmids encoding CHIKV P123 (CMV-P123-CHIKV), ONNV nsP4 fused at the C terminus to ubiquitin (CMV-ubinsP4-ONNV), and the polymerase-negative variant nsP4^GAA^ (CMV-ubinsP4^GAA^-ONNV), have been described previously (47, 55).

To generate *trans*-replicase variants harboring mutations in experimentally verified or predicted nsP4 interaction sites (Tables S1 and S2), mutations were introduced into CMV-ubinsP4-ONNV using either site-directed mutagenesis and standard subcloning procedures (for single substitutions) or synthetic DNA fragments (Genscript) followed by subcloning (for multiple substitutions). Selected mutations were also introduced into the matching viral genome context. For this purpose, the SP6 promoter in the SP6-CHIKV^ONNVnsP4^ icDNA clone (S. Wang, L. S. Lello, K. Rausalu, and A. Merits, unpublished data) was replaced with a CMV promoter to generate CMV-CHIKV^ONNVnsP4^, after which the wt fragments were replaced with mutated counterparts by restriction enzyme-based subcloning.

Three selected mutations were additionally introduced into pCMV-ICRES1, the infectious cDNA of CHIKV isolate LR-2006-OPY1 (East/Central/South African genotype) (56), using site-directed mutagenesis and subcloning. The resulting constructs were designated pCMV-CHIKV^F164A^, pCMV-CHIKV^K174S^, and pCMV-CHIKV^D393A^. Mutations corresponding to those in CHIKV^ONNVnsP4^ were also introduced into SFV6 using the infectious cDNA plasmid pCMV-SFV6 (57) via PCR-based site-directed mutagenesis and subcloning procedures. Final plasmid sequences were confirmed by Sanger sequencing and are provided in Data S1.

### *Trans*-replication assay

The *trans*-replication assay in U2OS cells was performed as previously described (47). Briefly, U2OS cells were seeded into 96-well plates at approximately 15,000 cells per well and co-transfected with 85 ng of CMV-CHIKV-P123 plasmid, 65 ng of CMV-ubinsP4-ONNV (wt or mutant) plasmid, and 85 ng of HSPolI-FG-CHIKV using Lipofectamine LTX with PLUS reagent (Thermo Fisher Scientific) according to the manufacturer’s instructions. For negative controls, the CMV-ubinsP4-ONNV plasmid was replaced with the polymerase-negative construct CMV-ubinsP4^GAA^-ONNV. Transfected cells were incubated at 37°C for 18 h. Following incubation, the cells were lysed, and Fluc and Gluc activities were measured using the Dual-Luciferase Reporter Assay System (Promega). Activities measured in cells expressing active replicases were normalized to those obtained from the corresponding control cells. All transfections were performed in triplicate, and each experiment was repeated at least three times.

### Virus rescue and titration

SFV6, CHIKV, and CHIKV^ONNVnsP4^ were rescued from the corresponding icDNA clones pCMV-SFV6, pCMV-ICRES1, and pCMV-CHIKV^ONNVnsP4^ as previously described (57). To rescue wt or mutant viruses, BHK-21 cells (8 × 10⁶) were transfected with 1 μg of icDNA by electroporation using a Bio-Rad Gene Pulser II unit (one pulse at 220 V and 975 μF) in 0.4-cm cuvettes (Thermo Fisher Scientific) and plated to 60 mm cell culture plates. Stocks of wt SFV6, CHIKV, CHIKV^ONNVnsP4^, and their mutant variants were collected at the onset of cytopathic effects (CPE) or at 72 h post-transfection, whichever occurred first. Supernatants were clarified by centrifugation at 3,000 × *g* for 10 min, and the virus titers were determined by plaque assay (SFV) or focus-forming assay (CHIKV, CHIKV^ONNVnsP4^). For plaque assays, serial dilutions of SFV6 or mutant virus stocks in GMEM containing 0.2% bovine serum albumin were used to infect confluent BHK-21 monolayers growing on 60-mm cell culture plates for 1 h at 37°C and 5% CO_2_. After adsorption, the inoculum was replaced with GMEM supplemented with 2% FBS and 0.8% carboxymethyl cellulose (CMC; Sigma Life Science). Following 72 h of incubation at 37°C, plaques were stained with crystal violet and counted. Focus-forming assays were performed on BHK-21 cells using a rabbit polyclonal antibody recognizing the capsid protein of CHIKV (prepared in-house). Viral titers were calculated using the following formula: PFU/mL = (average number of plaques/volume [mL] of virus added) × dilution factor.

An infectious center assay (ICA) was performed as described previously (58), except that BHK-21 cells were transfected with 5 µg of endotoxin-free plasmids pCMV-SFV6, pCMV-ICRES1, or pCMV-CHIKV^ONNVnsP4^. Mutants derived from these constructs were analyzed in the same manner. Tenfold serial dilutions were prepared using 10% transfected BHK-21 cells and seeded onto six-well tissue culture plates containing 1.5 × 10⁶ BHK-21 cells per well. After 2 h of incubation at 37°C, the medium was aspirated, and the cells were overlaid with 2 mL of growth medium supplemented with 0.8% CMC. After 2–3 days of incubation at 37°C, the plaques were visualized by crystal violet staining and counted, and the infectivity was expressed as PFU per microgram of plasmid DNA. All assays were performed three times.

### Western blotting

BHK-21 cells transfected with 5 μg of infectious cDNA plasmids were harvested at the same time that P0 virus stocks were collected. Briefly, after washing with 1 mL of PBS, the cells were lysed by boiling in 1× SDS sample buffer, and proteins were separated by SDS-PAGE using 10% polyacrylamide gels. The separated proteins were transferred onto polyvinylidene difluoride membranes and detected using antibodies against SFV or CHIKV capsid proteins (both in-house) and β-actin (sc-47778; Santa Cruz Biotechnology) or β-tubulin (AF2835; Beyotime) as loading controls. After incubation with appropriate secondary antibodies conjugated to fluorescent dyes (for CHIKV and CHIKV^ONNVnsP4^) or horseradish peroxidase (for SFV), the membranes were imaged using either the LI-COR Odyssey Fc imaging system or an ECL chemiluminescence assay.

### Multi-step growth curves

Confluent BHK-21 cells grown in 24-well plates were infected with P0 virus stocks at an MOI of 0.01. After a 1-h incubation with gentle rocking, the inoculum was removed, and the cells were overlaid with fresh GMEM supplemented with 2% FBS, followed by incubation at 37°C in a 5% CO₂ atmosphere. Supernatants were collected at 2, 4, 6, 8, 12, 24, and 36 hpi for SFV and 4, 8, 12, 24, and 48 hpi for CHIKV and CHIKV^ONNVnsP4^. Infections of *A. albopictus* C6/36 cells were performed similarly, except that cells were incubated at 28°C without supplemental CO_2_. Virus titers at each time point were determined by plaque assay or focus-forming assay on BHK-21 cells. All experiments were performed at least three times.

### Sequence analysis of mutant SFV, CHIKV^ONNVnsP4^, and CHIKV isolates

Plaque-purified SFV isolates were obtained as described previously (59). Briefly, BHK-21 cells in six-well plates were infected with 20 PFU of each analyzed mutant virus and overlaid with GMEM containing 2% FBS and 0.9% Bacto agar. At 48 hpi, a second overlay containing 0.9% agar and neutral red dye was added. After an additional 8 h of incubation, the plaques were picked and transferred into the growth medium of BHK-21 cells in 24-well plates. The cells were incubated at 37°C until CPE appeared, at which point viral stocks were collected. Genomic RNAs from six plaque-purified isolates per mutant were extracted using the RNeasy Minikit (Qiagen). RNAs were reverse-transcribed with random hexamers using the First-Strand cDNA Synthesis Kit (Thermo Fisher Scientific). The entire nonstructural region was PCR-amplified as overlapping fragments and analyzed by Sanger sequencing.

Analysis of mutations in CHIKV^ONNVnsP4^ and CHIKV mutants was carried out as previously described (60). Briefly, viral RNA extracted from each stock was reverse-transcribed, the region encoding nsP4 was PCR-amplified with ONNV-specific or CHIKV-specific primers, and the resulting products were cloned into the pJET1.2 vector (Thermo Fisher Scientific). From each library, six randomly selected clones were sequenced (Plasmidsaurus).

### Kunming mice and SFV infections

Six-week-old Kunming mice were housed under specific-pathogen-free and environmentally enriched conditions, with food and water provided *ad libitum*. In the experiment assessing viral attenuation, mice were inoculated intraperitoneally with 0.1 mL of PBSA (PBS containing 0.75% bovine serum albumin) containing 1,000 PFU of wt SFV6, SFV^F164A^, SFV^K174S^, or SFV^D393A^; control mice received PBSA alone (*n* = 8 per group). In a separate experiment, 6-week-old Kunming mice (*n* = 8 per group) were inoculated intracranially with 1,000 PFU of wt SFV6, SFV^F164A^, or 30 μL of PBSA. Animals were monitored twice daily for up to 14 days for clinical signs, and mice that reached defined humane endpoints were euthanized, after which their brains were collected for viral titration.

For the determination of viremia and neuroinvasiveness, mice were infected with 1,000 PFU of wt SFV6, SFV^F164A^, SFV^K174S^, or SFV^D393A^ (*n* = 8 per group) as described above. Blood samples were collected on PID 1 and 3, after which the animals were euthanized and brains were harvested. Brains were homogenized in PBSA, and the virus titers in brain homogenates and serum samples were determined by plaque assay on BHK-21 cells.

For analysis of neutralizing antibody responses, 6-week-old Kunming mice (*n* = 5 per group) were immunized intraperitoneally with 10⁴, 10⁵, or 10⁶ PFU of SFV^F164A^; control mice received MEM. Blood samples were collected at 2 weeks post-immunization. For analysis of protective immunity, 4-week-old Kunming mice (*n* = 5 per group) were immunized intraperitoneally with 10⁴, 10⁵, or 10⁶ PFU of SFV^F164A^; control mice (*n* = 10) received MEM. After 2 weeks, mice were challenged intraperitoneally with 1,000 PFU of wt SFV6. Of the control mice, five were challenged with wt SFV6 and five were mock-infected. Animals were monitored twice daily for up to 14 days for clinical signs, and mice reaching predefined humane endpoints were euthanized.

### Plaque reduction neutralization tests

Serial dilutions of sera were prepared in DMEM containing 2% FBS. The starting dilution was 1:10, and the maximum dilution for each serum sample was 1:160. Diluted sera (200 μL) were incubated with an equal volume of the SFV6 stock containing 750 PFU/mL at 37°C for 1 h; in the blank control, virus samples were incubated with DMEM containing 2% FBS. Following incubation, the mixtures were used to infect BHK-21 cell monolayers grown in 12-well plates. After a 1-h adsorption period at 37°C, the inoculum was removed, and the cells were overlaid with medium containing 1% CMC. After 48 h, the overlay was aspirated, and the plaques were stained with crystal violet and counted.

### C57BL/6J mice and CHIKV infections

For CHIKV infection experiments, 6- to 8-week-old C57BL/6J mice, with comparable sex and age distributions across groups, were inoculated subcutaneously on the ventral side of the foot with 10,000 PFU of wt CHIKV, CHIKV^F164A^, CHIKV^K174S^, or CHIKV^D393A^ (*n* = 7 in the first experiment and *n* = 4 in the second experiment) diluted in PBS to a final volume of 50 μL, as described previously (61–64). Mock-infected animals received PBS alone. In the first experiment, disease score (assessed by foot swelling) and body weight were monitored daily for 10 days. Blood samples were collected at PID 1, PID 5, and PID 7. The serum was separated and stored at −80°C until titration. In the second experiment, hind-limb ankle joints and quadriceps muscles were collected at PID 7 for viral titration and histological analysis, respectively.

### Plaque assay of viruses from CHIKV-infected animals

Vero E6 cells were seeded in 12-well plates at 2 × 10^5^ cells per well and cultured overnight at 37°C in DMEM supplemented with 5% FBS. Serum samples and clarified ankle-joint homogenates were serially diluted and added to Vero E6 cell monolayers. After virus adsorption, the cells were overlaid with 1.2% colloidal microcrystalline cellulose (Sigma-Aldrich) and incubated for 72 h at 37°C. The cells were then fixed with 4% paraformaldehyde (PFA) and stained with 0.1% crystal violet.

### H&E staining and analysis

Quadriceps muscle tissues were fixed in 4% PFA, embedded in paraffin, sectioned at a thickness of 5 μm, and stained with H&E. Stained slides were scanned using an EVIDENT VS200 Slide Scanner, and inflammatory-cell infiltrates were quantified using QuPath.

### Statistical analysis

Statistical analyses were performed using GraphPad Prism software. Data were evaluated using the Kruskal-Wallis test, except virus growth curves, which were analyzed using repeated-measures ANOVA, and *in vivo* survival curves, which were analyzed using the log-rank (Mantel-Cox) test. *P*-values of ≤ 0.05 (*), ≤0.01 (**)*,* ≤0.001 (***), and ≤0.0001 (****) were used to indicate the levels of significance for comparisons between mutant and wt samples. Each experiment was repeated to obtain a minimum of three independent biological replicates.

## SUPPLEMENTAL MATERIAL

Data S1 mbio.01638-26-s0001.xlsx Final plasmid sequences confirmed by Sanger sequencing.

Supplemental legends mbio.01638-26-s0002.docx Supplemental table and data legends.

Table S1 mbio.01638-26-s0003.docx Targeted interactions within the replicase core, introduced mutations, and their expected effects.

Table S2 mbio.01638-26-s0004.docx Mutations targeting nsP4:RNA interactions and their expected effects.

Table S3 mbio.01638-26-s0005.docx Properties of viruses harboring mutations in nsP4.

Table S4 mbio.01638-26-s0006.docx Potential compensatory changes found in the SFV mutants.

ASM does not own the copyrights to Supplemental Material that may be linked to, or accessed through, an article. The authors have granted ASM a non-exclusive, world-wide license to publish the Supplemental Material files. Please contact the corresponding author directly for reuse.
