# Supplementary material for: Targeting conserved interaction surfaces of alphavirus nsP4 supports the rational design of live-attenuated vaccine candidates
Source: mBio. 2026 Sep 10;17(10):e01638-26. doi: 10.1128/mbio.01638-26 (PMC13712852; doi:10.1128/mbio.01638-26)
Supplement: Supplementary file 1 [file mbio.01638-26-s0002.docx]

**Table S1. Targeted interactions within the replicase core, introduced mutations and their expected effects.**

**Table S2. Mutations targeting nsP4:RNA interactions and their expected effects.**

**Table S3. Properties of viruses harboring mutations in nsP4.**

**Table S4.** **Potential compensatory changes found in the SFV mutants.**

**Data S1. Final plasmid sequences confirmed by Sanger sequencing.**
