# Supplementary material for: Targeting conserved interaction surfaces of alphavirus nsP4 supports the rational design of live-attenuated vaccine candidates
Source: mBio. 2026 Sep 10;17(10):e01638-26. doi: 10.1128/mbio.01638-26 (PMC13712852; doi:10.1128/mbio.01638-26)
Supplement: Supplementary file 2 [file mbio.01638-26-s0003.docx]

**Table S1. Targeted interactions within the replicase core, introduced mutations and their expected effects.**

| **Interaction partner** | **Mutation(s) in nsP4** | **Domain** | **Expected impact** |
| --- | --- | --- | --- |
| ***nsP1*** | V27A+E28A* | NTD | Destabilize the folding of nsP4 NTD and its interaction with nsP1;  compromise the replicase activity. |
|  | K114A+N117A* | Beginning of fingers | Destabilize the folding of nsP4 NTD and its interaction with nsP1;  compromise the replicase activity. |
|  | Y139A | Fingers | Destabilize the folding of nsP4 NTD;  compromise the replicase activity. |
|  | S155A | Fingers | Destabilize the interaction with nsP1;  compromise the replicase activity. |
|  | S157A | Fingers | Similar to S155A. |
|  | C158A | Fingers | Similar to S155A |
|  | F164A | Fingers | Destabilize the folding of nsP4 NTD and its interaction with nsP1;  compromise the replicase activity. |
|  | P166A | Fingers | Destabilize the folding of nsP4 NTD and its interaction with nsP1;  compromise the replicase activity. |
|  | L169A | Fingers | Destabilize the folding of nsP4 NTD and its interaction with nsP1;  compromise the replicase activity. |
|  | E236A | Palm | Destabilize the interaction with nsP1;  compromise the replicase activity. |
|  | M283A | Fingers | Destabilize the folding of nsP4 NTD;  compromise the replicase activity. |
|  | E304A | Fingers | Destabilize the folding of nsP4 NTD and its interaction with nsP1;  compromise the replicase activity. |
|  | R306A | Fingers | Destabilize the folding of nsP4 NTD and its interaction with nsP1;  compromise the replicase activity (more than E304A). |
|  | Q577A | Thumb | Destabilize the interaction with nsP1;  compromise the replicase activity. |
|  | V27A+E28A+K114A+N117A | Combination of V27A+E28A and K114A+N117A | |
|  | V27A+E28A+Y139A | Combination of V27A+E28A and Y139A | |
|  | 155-169 | Combination of S155A, S157A, C158A, F164A, P166A and L169A | |
|  | V27A+E28A+E236A | Combination of V27A+E28A and E236A | |
|  | V27A+E28A+M283A | Combination of V27A+E28A and M283A | |
|  | E304A+R306A | Combination of E304A and R306A | |
|  | V27A+E28A+Q577A | Combination of V27A+E28A and Q577A | |
|  | E236A+M283A+Q577A | Combination of E236A, M283A and Q577A | |
| ***nsP2*** | D219A | Palm | Interacts with R101 of nsP2; mutation destabilize the interaction with nsP2 and compromise the replicase activity. |
|  | E226A | Palm | Interacts with S54 of nsP2; mutation destabilize the interaction with nsP2 and compromise the replicase activity. |
|  | E392A | Palm | Interacts with residues A57-R59 of nsP2;  mutation destabilize the interaction with nsP2 and compromise the replicase activity. |
|  | D393A | Palm | Interacts with R56 of nsP2; mutation destabilize the interaction with nsP2 and compromise the replicase activity. |
|  | D402A | Palm | Interacts with R59 of nsP2; mutation destabilize the interaction with nsP2 (possibly also with nsP1) and compromise the replicase activity. |
|  | E405A | Palm | Interacts with R59 of nsP2; mutation destabilize the interaction with nsP2 (possibly also with nsP1) and compromise the replicase activity. |
|  | D219A+E226A | Combination of D219A and E226A | |
|  | E392A+D393A | Combination of E392A and E393A | |
|  | E226A+D402A | Combination of E226A and D402A | |
|  | D402A+E405A | Combination of D402A and E405A | |

* these mutations were not analyzed individually
