# Supplementary material for: Targeting conserved interaction surfaces of alphavirus nsP4 supports the rational design of live-attenuated vaccine candidates
Source: mBio. 2026 Sep 10;17(10):e01638-26. doi: 10.1128/mbio.01638-26 (PMC13712852; doi:10.1128/mbio.01638-26)
Supplement: Supplementary file 3 [file mbio.01638-26-s0004.docx]

**Table S2. Mutations targeting nsP4:RNA interactions and their expected effects.**

| **Mutation(s) in nsP4** | **Domain** | **Expected impact/rational** |
| --- | --- | --- |
| del1-15 (deletion of aa 1-15) | NTD | Coordination of RNA |
| del9-10 (deletion of Q9+G10)* | NTD | Coordination of RNA |
| 58-73 (replacement of aa 58, 60, 61, 62, 63, 65, 66, 67, 68, 70, 71, and 73 with Val)* | NTD | Coordination of RNA |
| T163W | Index finger (RNA pore, pore center) | Import/export of RNA via the pore |
| K168S | Loop (RNA pore) | Import/export of RNA via the pore |
| R170S | Loop (RNA pore) | Import/export of RNA via the pore |
| K174S | Loop (RNA pore) | Import/export of RNA via the pore |
| C207S | Index finger flexible loop (RNA pore, upper region) | Import/export of RNA via the pore |
| V209K | Index finger flexible loop (RNA pore, upper region) | Import/export of RNA via the pore |
| Q211A | Index finger flexible loop (RNA pore, upper region) | Import/export of RNA via the pore |
| K261A | Pinky finger (major RNA groove) | Disturbs interaction with RNA? |
| K264A | Pinky finger (major RNA groove) | Disturbs interaction with RNA? |
| R285A | Middle finger (RNA pore, pore center) | Affects putative RNA channel |
| R285D |  | Affects putative RNA channel |
| H327A | Palm (after middle finger) | Affects NTP incorporation? |
| R328A | Palm (after middle finger) | Affects NTP incorporation? |
| S412V | Ring finger (RNA pore, pore center) | Affects putative RNA channel |
| R421V | Ring finger (RNA pore, pore center) | Affects putative RNA channel |
| K423V | Ring finger (RNA pore, pore center) | Affects putative RNA channel |
| K611A | C-terminus | Disturbs interaction with RNA? |
| delGGPK (deletion of aa 608-611) | C-terminus | To reveal functional importance of CTD tail of nsP4 |
| K168S+R170S+K174S | Combination of K168S, R170S, and K174S | |
| C207S+V209K+Q211A | Combination of C207S, V209K, and Q211A | |
| H327A+R328A | Combination of H327A and R328A | |
| H327E+R328E* | Combination of H327E and R328E | |
| S412V+R421V+K423V | Combination of S412V, R421V, and K423V | |

* these mutations were not analyzed individually. CTD – C-terminal domain of RdRp.
