# Supplementary material for: Targeting conserved interaction surfaces of alphavirus nsP4 supports the rational design of live-attenuated vaccine candidates
Source: mBio. 2026 Sep 10;17(10):e01638-26. doi: 10.1128/mbio.01638-26 (PMC13712852; doi:10.1128/mbio.01638-26)
Supplement: Supplementary file 4 [file mbio.01638-26-s0005.docx]

**Table S3. Properties of viruses harboring mutations in nsP4.**

| **Interaction sites** | **Mutations in nsP4** | **CHIKV^ONNV^** | | **SFV** | |
| --- | --- | --- | --- | --- | --- |
|  |  | ***ICA**** | ***P0 titer***** | ***ICA*** | ***P0 titer*** |
| ***Parental*** | None | 1.14 × 10^6^ | 1.3 × 10^8^ | 5.3 × 10^4^ | 6.3 × 10^8^ |
| ***nsP1*** | Y139A | 1.2 × 10^5^ | 3.8 × 10^7^ | 2.2 × 10^3^ | 2.3 × 10^8^ |
|  | F164A | 3.3 × 10^5^ | 1.9 × 10^7^ | 6.0 × 10^4^ | 5.0 × 10^7^ |
|  | E304A | 2.9 × 10^5^ | 1.9 × 10^7^ | 1.3 × 10^3^ | 5.0 × 10^8^ |
|  | R306A | 1.7 × 10^5^ | 9.3 × 10^6^ | 1.1 × 10^3^ | 1.8 × 10^8^ |
| ***nsP2*** | D219A | 3.3 × 10^5^ | 1.6 × 10^7^ | 3.9 × 10^4^ | 1.3 × 10^8^ |
|  | E392A | 4.1 × 10^5^ | 1.6 × 10^7^ | 2.0 × 10^3^ | 1.0 × 10^7^ |
|  | D393A | 3.6 × 10^5^ | 2.0 × 10^7^ | 9.2 × 10^3^ | 1.4 × 10^8^ |
| ***RNA*** | S412V+R421V+K423V | 1.2 × 10^4^ | 2.3 × 10^6^ | 0 | 0 |
|  | V209K | 1.4 × 10^5^ | 9.3 × 10^6^ | 1.5 × 10^4^ | 1.8 × 10^8^ |
|  | Q211A | 1.1 × 10^6^ | 2.5 × 10^7^ | 3.7 × 10^3^ | 4.0 × 10^8^ |
|  | K174S | 1.9 × 10^6^ | 1.6 × 10^7^ | 2.3 × 10^4^ | 1.0 × 10^7^ |
|  | C207S | 0 | 1.0 × 10^7^ | 0 | 1.0 × 10^7^ |
|  | K168S+R170S+K174S | 0 | 0 | 0 | 0 |
|  | C207S+V209K+Q211A | 0 | 0 | 0 | 0 |
|  | K264A | 0 | 0 | 0 | 0 |
|  | H327A | 0 | 0 | 1.0 × 10^1^ | 3.0 × 10^2^ |

* ICA, infectious center assay; values are expressed as PFU/μg of transfected icDNA plasmid

**P0, virus stock harvested from transfected cells; titers are expressed in FFU/mL for CHIKV^ONNVnsP4^ derived viruses and PFU/mL for SFV-derived viruses
