# Supplementary material for: Targeting conserved interaction surfaces of alphavirus nsP4 supports the rational design of live-attenuated vaccine candidates
Source: mBio. 2026 Sep 10;17(10):e01638-26. doi: 10.1128/mbio.01638-26 (PMC13712852; doi:10.1128/mbio.01638-26)
Supplement: Supplementary file 5 [file mbio.01638-26-s0006.docx]

**Table S4.** **Potential compensatory changes found in the SFV mutants**

| **Mutation** | **Putative compensatory changes** | | |
| --- | --- | --- | --- |
|  | ***Pseudoreversions*** | ***Second site changes in nsP4*** | ***Other nsPs*** |
| Y139A | None | None | None |
| F164A | None | None | None |
| E304A | None | None | None |
| R306A | None | F191L (1)* | nsP2 R623K (1) |
| D219A | None | None | None |
| E392A | None | None | None |
| D393A | None | None | nsP3 A137T (1) |
| K174S | None | H176R (4)  E316K (1)  H176R+E316K (1) | None |
| C207S | C207S to C207F (6) | None | None |
| V209K** | V209K to V209T and  V209K to V209I and | E481K (2)  E481K (1)***  - | nsP2 E399G (1)  nsP3 S183N +D390E (1) |
| Q211A | None | None | nsP3 D390E (1)  nsP3 N126I (1) |
| H327A | None | Q192R (4)  V187I (1)  V1871 and | nsP3 S422I (1) |

* Numbers in parentheses indicate the number of isolates in which the mutation or combination of mutations was detected.

** In one isolate of this virus, no additional mutations were detected.

*** Pairs or triplets of mutations highlighted in gray were detected within the same viral isolate.
